# Supplementary material for: Hydroxyurea-Mediated Cytotoxicity Without Inhibition of Ribonucleotide Reductase
Source: Cell Rep. 2016 Nov 1;17(6):1657–70. doi: 10.1016/j.celrep.2016.10.024 (PMC5134839; doi:10.1016/j.celrep.2016.10.024)
Supplement: Document S1. Figures S1–S5 and Tables S1–S4 [file mmc1.pdf]

Cell Reports, Volume *17*

Supplemental Information

Hydroxyurea-Mediated Cytotoxicity

Without Inhibition of Ribonucleotide Reductase

Li Phing Liew, Zun Yi Lim, Matan Cohen, Ziqing Kong, Lisette Marjavaara, Andrei Chabes, and Stephen D. Bell

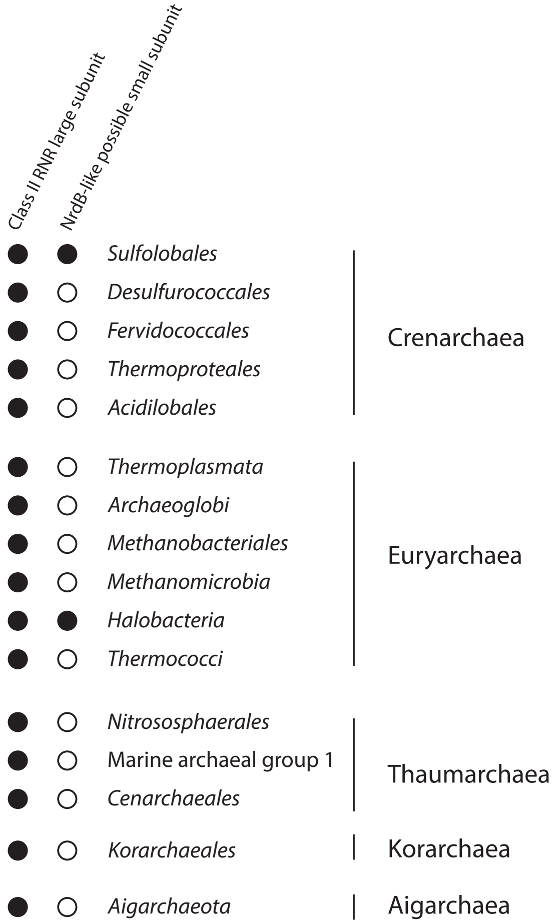

Figure S1. Distribution of Class II Ribonucleotide Reductase large subunits and homologs of the NrdB Class I small subunit in the indicated groups of archaea (related to Figure 1). Filled circles indicate presence of homologs, open circles indicate their absence.

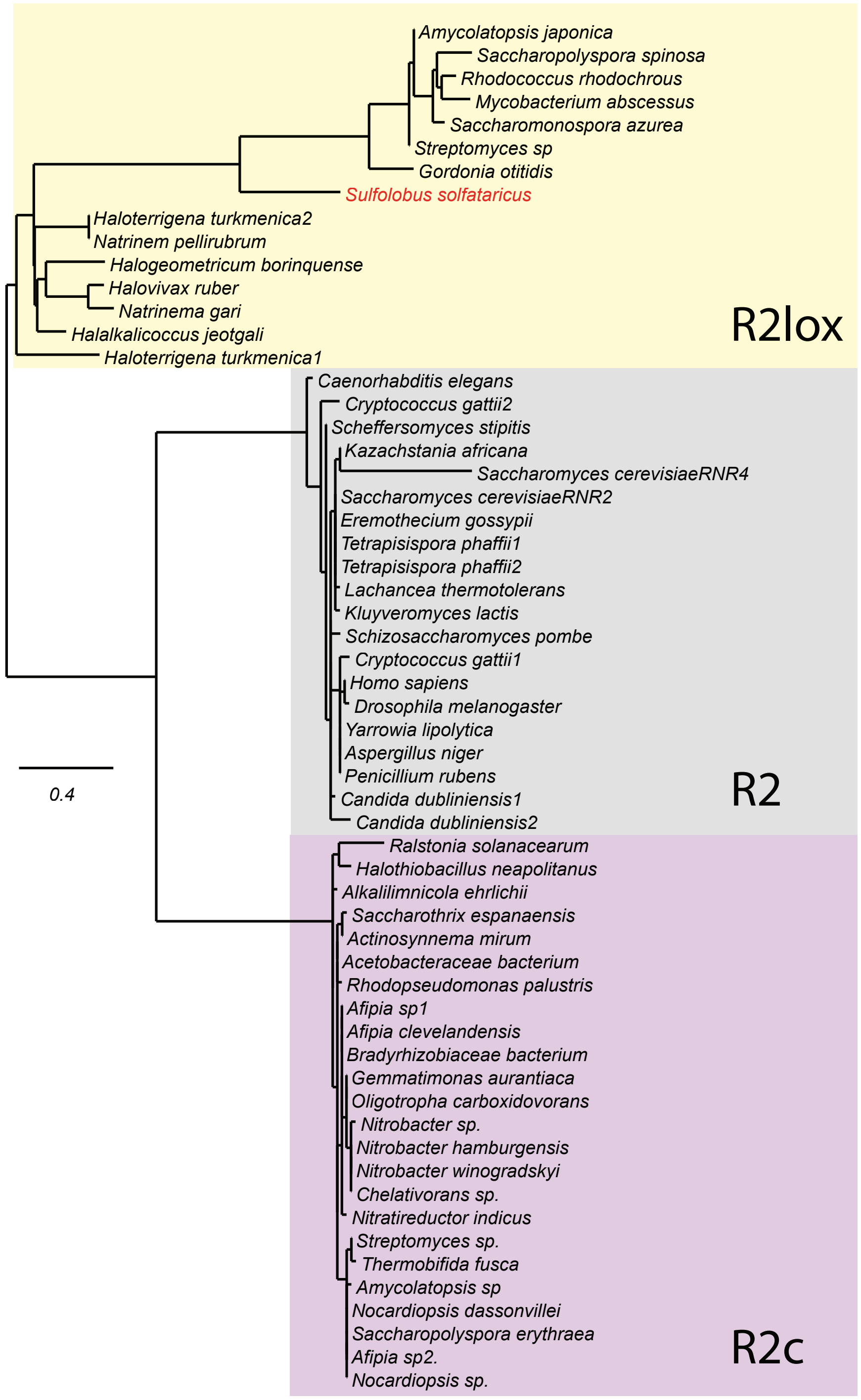

Figure S2. *Sulfolobus* "NrdB" is not a bona fide RNR-subunit but rather is a R2lox family member (related to Figure 1). Phylogenetic analyses of homologs of the NrdB-like protein reveal that the *Sulfolobus* SSO2498 gene product (red) is a member of the R2lox family of non-RNR-associated ligand-dependent oxidases (Andersson and Hogbom, 2009; Hogbom, 2011). The tree was generated using the server at www.phylogeny.fr (Dereeper et al., 2008). Accession numbers are listed in Table S4.

```
SSO2498        1 ------------------------------------------------------------
Ecoli          1 -MAYTTF-----------------------------------------------------
Pseudomonas    1 MLSWDEFDEEDTTTAAPATAAAQPASQTAVKLDNEAAGSVEEARAVAADDSDAIARAKRA

SSO2498        1 -----MGMSFE---------EYKHEYFKSIRS-----GGLNWSLFPMKLYQLGKKLFWDP
Ecoli          7 ------SQ--------TKNDQLKEPMFFGQPVNVARYDQQKY-DIFEKLIEKQLSFFWRP
Pseudomonas   61 LDELDIREGLNELEGEAARVRVDEKRMINARADLNQLVPFKY-DWAWQKYLDGCANHWMP

SSO2498       42 ANIDLSKDAEDWKKLNDLE----KMFIINVGSKFAAGEEAVALDLHPLIVTLVKEGRVEE
Ecoli         52 EEVDVSRDRIDY---QALPEHEKHIFISNLKYQ-TLLDSIQGRSP--NVALLPLISIPEL
Pseudomonas  120 QEVNMNADIALWKSKDGLSEDERRIVKRNLGFF-STADSLVANNL--VLATYRLITNPEC

                                   *
SSO2498       98 VMYLEQFVYEESKHVEAFRRFFDIVN-----------VMEDLSAYTKDLSPNYKKIFYEE
Ecoli        106 ETWVETWAFSETIHSRSYTHIIRNIVNDPSVVFDDIVTNEQIQKRAEGISS-----YYDE
Pseudomonas  177 RQYILRQAFEEAIHTHAYQYCIESLGMDEGEIFNMYHEIPSVAKK---------------

SSO2498      147 LP--KAMWNLSRDPS------------PE---NQVRAVVTYNLIVEGVAAEGGYNIFRQI
Ecoli        161 LIEMTSYWHLLGEGTHTVNGKTVTVSLRELKKKLY-LCLMSVNALEAIRFYVSFACSF-A
Pseudomonas  222 -----ASWGLKYT--RSISDPTFQTGTPETDKQFLKNLIAYYCVLEGIFFYCGFTQIL-S

SSO2498      190 TNTRKILPGLAKMVNLIATDESRHIAFGIYLITRLVKEYGEGV------------YKAAM
Ecoli        219 FAERELMEGNAKIIRLIARDEALHLTGTQHMLNLLRSGADDPEMAEIAEECKQECYDLFV
Pseudomonas  274 MGRRNKMTGTAEQFQYILRDESMHLNFGIDMINQIKIENPHLWDA----EMKDEATQMIL

SSO2498      238 DHINYLAPYAIGIFSEPTMPQVESLPFNLTNIELVDYAKKLLNTRIDAINRARRMKLEML
Ecoli        279 QAAQQEKDW-----ADYLFRDGSMI--GLNKDILCQYVEYITNIRMQAVGLDL------P
Pseudomonas  330 QGTQLEIEY-----ARDTMP-RGVL--GMNAAMMEDYLKFIANRRLTQIGLKE------E

SSO2498      298 LPKD---LDVIESW-------------------------------------
Ecoli        326 FQTRSNPIPWINTWLVSDNVQVAPQEVEVSSYLVGQIDSEVDTDDLSNFQL
Pseudomonas  376 YPGTTNPFPWMSEIMDLKKEKNF-FETRVIEYQTGGALS---WD-------
```

Figure S3 The *Sulfolobus* NrdB-related protein lacks the residue that forms the tyrosyl radical in true Class I RNR small subunits (related to Figure 1). Sequence comparison of *Pseudomonas aeruginosa* and *E. coli* NrdB with the *S. solfataricus* NrdB-like protein SSO2498. The essential tyrosine residue in the bacterial proteins is highlighted in red indicating its substitution by phenylalanine in the archaeon. Proteins sequences were aligned using ClustalOmega (Sievers et al., 2011) and shaded using the BoxShade Server (http://www.ch.embnet.org/software/BOX_form.html)

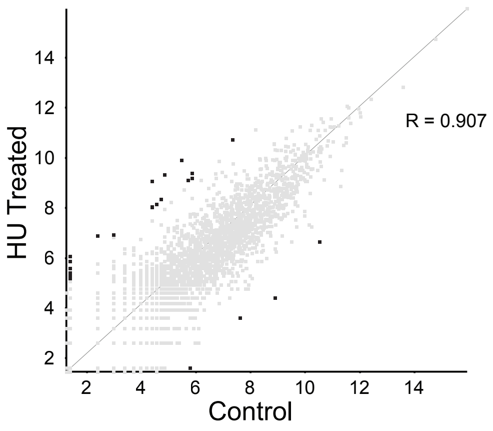

Figure S4. Comparison of RNA Seq data from control and treated cells (related to Table 1). Scatter plot comparing RNA-Seq results from HU-treated and untreated *S. solfataricus* cells. Statistically significant changes in level are indicated in black.

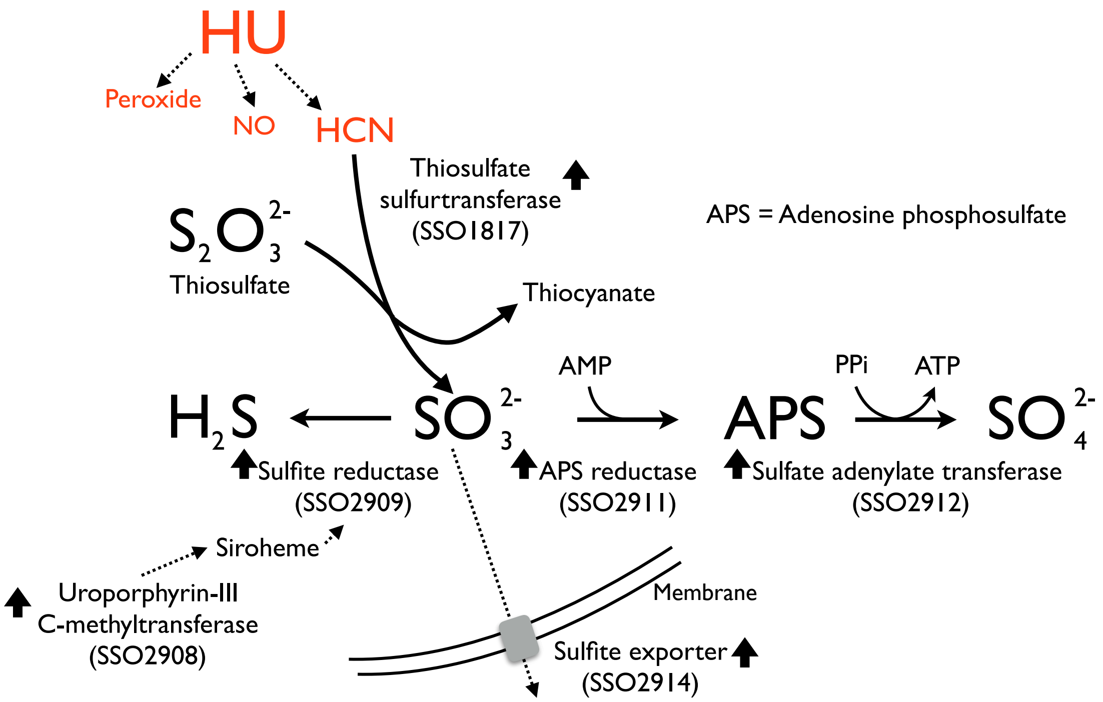

Figure S5. Potential detoxification pathway for HU breakdown products inferred from RNA Seq data (related to Table 1). RNA Seq data reveal elevated levels of transcripts for the enzymes indicated by the bold black arrows. We propose that cyanide generated by the thermal breakdown of HU is fed into this pathway by the action of thiosulfate sulfurtransferase generating thiocyanate and sulfite.

Gene	mRNA ratio (HU treated:untreated)
orc1-1	0.25
orc1-2	1.7
orc1-3	0.62
whiP	1.1
priL	0.64
radA	2.4
mre11	2.3

Table S1:Related to Figure 2: qRT-PCR quantitation of the indicated transcripts, comparing cells treated with 0 and 5 mM HU for 4 hours.

Oligonucleotide	Sequence (5' – 3')
Orc1-1fwd	GGGAAAGACAGCCGTAGTGA
Orc1-1rvs	AACCCGGTAAATGGAACCTT
Orc1-2fwd	GGATCGCCAGTGGTATTGTT
Orc1-2rvs	CCCATTGTGATCTCATCAGC
Orc1-3fwd	AGCACGGTGATGCAAGGAAAGCA
Orc1-3rvs	CACTAACGTGTCAACCTCGTCAAGA
WhiPfwd	CCCAGATCCAAATTGGTTGA
WhiPrvs	CGCTTAAGTGCCATCTTCCT
RadAfwd	TGGCCGTAAGGCAACAAAAGCT
RadArvs	ACCACCAACTGCCACAGTAGGGT
Mre11fwd	ACCACGACACTCCAAAGAGG
Mre11rvs	GCGGATAACGCGCTAACTAA
PriLfwd	TGAACCCGTACTCGTGTTTT
PriLrvs	TCTCCTCATTCTCGGTGTGA

Table S2, related to Figure 2: Primer pairs used for the qRT-PCR analyses.

Oligonucleotide	Sequence (5' – 3')
Sso1521fwd	GGCAAGTGGGTTGTTGAGAT
Sso1521rvs	TTACAGCGTCCCAAAAGTCC
Sso2661fwd	GGAGCCGCAGCAGTATTAAG
Sso2661rvs	ACGCATACCCTGAAAGATCG
OriC1fwd	TTTACCAGAGACCTACCCCATTGTTTC
OriC1rvs	GGGACCCATCTATTTCCTCTGG
OriC2fwd	TGTTTCAGATGAAATTAAGAGAGTGTCTC
OriC2rvs	CCCAGTTATGCAGTAAATTTCCTGTG
OriC3fwd	GTTATACTTCTATGGGCATATTGGGG
OriC3rvs	TGACATTAAATTCTCCCTCCTACTTGC
Sso2847fwd	GGGAAGCACTAAGCCAGATAATCC
Sso2847rvs	ACCTGAAATGCAGCTGGAGG

Table S3: Related to Figure 4. Primers used for amplifying the loci indicated in their names. The Sso1521 and Sso2661 yielded amplicons that were used for preparation of probes for 2D gel analyses. The remaining primers were used in qPCR quantitation of DNA recovered in ChIP experiments.

Accession Number	Species
AAK42633.1	*Sulfolobus solfataricus P2*
WP_008415238.1	*Halalkalicoccus jeotgali*
WP_006054776.1	*Halogeometricum borinquense*
WP_012945201.1	*Haloterrigena turkmenica ORF1*
WP_012945997.1	*Haloterrigena turkmenica ORF2*
WP_015298815.1	*Natrinema pellirubrum*
WP_008456675.1	*Natrinema gari*
AGB17208.1	*Halovivax ruber*
WP_052617888.1	*Mycobacterium abscessus*
WP_054370857.1	*Rhodococcus rhodochrous*
WP_005448065.1	*Saccharomonospora azurea*
WP_010308649.1	*Saccharopolyspora spinosa*
WP_007238417.1	*Gordonia otitidis*
WP_038507879.1	*Amycolatopsis japonica*
WP_009083906.1	*Streptomyces sp. AA4*
ABI57445.1	*Alkalilimnicola ehrlichii*
EHL99301.1	*Acetobacteraceae bacterium*
WP_012823881.1	*Halothiobacillus neapolitanus*
WP_011582020.1	*Chelativorans sp. BNC1*
EKS35359.1	*Afipia clevelandensis*
EGP10090.1	*Bradyrhizobiaceae bacterium SG-6C*
WP_009340611.1	*Afipia sp.1*
WP_009451442.1	*Nitratireductor indicus*
WP_013224744.1	*Afipia sp.2*
ABD89058.1	*Rhodopseudomonas palustris*
WP_009075962.1	*Streptomyces sp. AA4*
WP_016333204.1	*Amycolatopsis sp.*
ACI93866.1	*Oligotropha carboxidovorans*
WP_009947973.1	*Saccharopolyspora erythraea*
ABA06137.1	*Nitrobacter winogradskyi*
CCH32194.1	*Saccharothrix espanaensis*
BAH38790.1	*Gemmatimonas aurantiaca*
ABE64392.1	*Nitrobacter hamburgensis*
WP_014908832.1	*Nocardiopsis sp.*
WP_009796979.1	*Nitrobacter sp.*
ACU37069.1	*Actinosynnema mirum*
ADH69995.1	*Nocardiopsis dassonvillei*
WP_011293419.1	*Thermobifida fusca*
NP_982406.1	*Eremothecium gossypii*
XP_001401310.1	*Aspergillus niger*
NP_497821.1	*Caenorhabditis elegans*
XP_002418255.1	*Candida dubliniensis1*

XP_002418662.1	*Candida dubliniensis2*
XP_003196606.1	*Cryptococcus gattii1*
XP_003196693.1	*Cryptococcus gattii2*
XP_003959265.1	*Kazachstania africana*
XP_455757.1	*Kluyveromyces lactis*
XP_002551960.1	*Lachancea thermotolerans*
XP_002567793.1	*Penicillium rubens*
XP_001382334.2	*Scheffersomyces stipitis*
WP_003273165.1	*Ralstonia solanacearum*
NP_596546.1	*Schizosaccharomyces pombe*
XP_003685845.1	*Tetrapisispora phaffii1*
XP_003688271.1	*Tetrapisispora phaffii2*
XP_500466.1	*Yarrowia lipolytica*
AJR59499.1	*Saccharomyces cerevisiae RNR2*
NP_001025	*Homo sapiens*
NP_525111.1	*Drosophila melanogaster*
AJR77267.1	*Saccharomyces cerevisiae RNR4*

Table S4 Related to Figures 1 and S2: Accession numbers of proteins sequences used in the phylogenetic tree in Figure S2.

Additional References

Dereeper, A., Guignon, V., Blanc, G., Audic, S., Buffet, S., Chevenet, F., Dufayard, J.F., Guindon, S., Lefort, V., Lescot, M., Claverie, J.M. and Gascuel, O. (2008) Phylogeny.fr: robust phylogenetic analysis for the non-specialist. Nucleic Acids Res. *36*, W365-369

Sievers, F., Wilm, A., Dineen, D., Gibson, T.J., Karplus, K. ,Li, W., Lopez, R., McWilliam, H., Remmert, M., Söding, J., Thompson, J.D. and Higgins, D.G. (2011) Fast, scalable generation of high-quality protein multiple sequence alignments using Clustal Omega. Mol. Syst. Biol. *7,* 539
