# Supplementary material for: Hydroxyurea-Mediated Cytotoxicity Without Inhibition of Ribonucleotide Reductase
Source: Cell Rep. 2016 Nov 1;17(6):1657–70. doi: 10.1016/j.celrep.2016.10.024 (PMC5134839; doi:10.1016/j.celrep.2016.10.024)
Supplement: Document S2. Article plus Supplemental Information [file mmc2.pdf]

Cell Reports

Article

Hydroxyurea-Mediated Cytotoxicity Without Inhibition of Ribonucleotide Reductase

Graphical Abstract

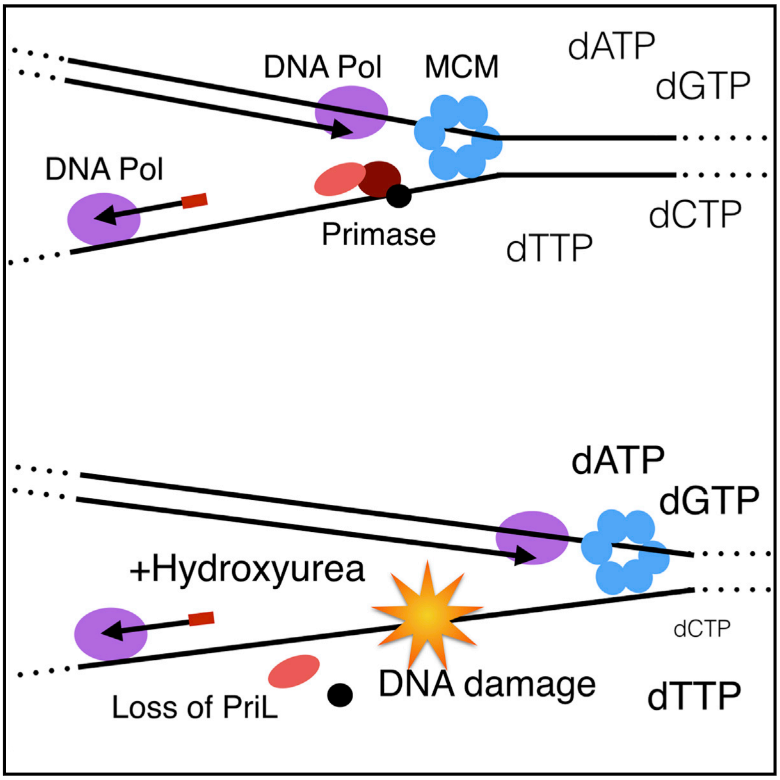

Authors

Li Phing Liew, Zun Yi Lim, Matan Cohen, Ziqing Kong, Lisette Marjavaara, Andrei Chabes, Stephen D. Bell

Correspondence

stedbell@indiana.edu

In Brief

Liew et al. demonstrate that hydroxyurea (HU) is toxic to the hyperthermophilic archaeon *Sulfolobus solfataricus*. Although archaea lack the classical class I ribonucleotide reductase target of HU, Liew et al., demonstrate HU causes impaired DNA replication in *Sulfolobus*, correlating with loss of the regulatory subunit of DNA primase.

Highlights

- *Sulfolobus* has a HU-insensitive class II ribonucleotide reductase
- HU impairs DNA replication and is toxic to *Sulfolobus* cells
- HU treatment leads to selective loss of the regulatory subunit of DNA primase

Accession Numbers

GSE87612

Liew et al., 2016, Cell Reports *17*, 1657–1670
November 1, 2016 © 2016 The Author(s).
http://dx.doi.org/10.1016/j.celrep.2016.10.024

OPEN ACCESS
CellPress

Hydroxyurea-Mediated Cytotoxicity Without Inhibition of Ribonucleotide Reductase

Li Phing Liew,[1] Zun Yi Lim,[2] Matan Cohen,[3] Ziqing Kong,[4] Lisette Marjavaara,[4] Andrei Chabes,[4,5] and Stephen D. Bell[2,3,6,*]
[1]Sir William Dunn School of Pathology, South Parks Road, Oxford OX1 3RE, UK
[2]Department of Molecular and Cellular Biochemistry, Indiana University, Simon Hall MSB, 212 South Hawthorne Drive, Bloomington, IN 47405, USA
[3]Department of Biology, Indiana University, Simon Hall MSB, 212 South Hawthorne Drive, Bloomington, IN 47405, USA
[4]Department of Medical Biochemistry and Biophysics, Umeå University, SE 90197 Umeå, Sweden
[5]Laboratory for Molecular Infection Medicine Sweden, Umeå University, SE 90197 Umeå, Sweden
[6]Lead Contact
*Correspondence: stedbell@indiana.edu
http://dx.doi.org/10.1016/j.celrep.2016.10.024

SUMMARY

In many organisms, hydroxyurea (HU) inhibits class I ribonucleotide reductase, leading to lowered cellular pools of deoxyribonucleoside triphosphates. The reduced levels for DNA precursors is believed to cause replication fork stalling. Upon treatment of the hyperthermophilic archaeon *Sulfolobus solfataricus* with HU, we observe dose-dependent cell cycle arrest, accumulation of DNA double-strand breaks, stalled replication forks, and elevated levels of recombination structures. However, *Sulfolobus* has a HU-insensitive class II ribonucleotide reductase, and we reveal that HU treatment does not significantly impact cellular DNA precursor pools. Profiling of protein and transcript levels reveals modulation of a specific subset of replication initiation and cell division genes. Notably, the selective loss of the regulatory subunit of the primase correlates with cessation of replication initiation and stalling of replication forks. Furthermore, we find evidence for a detoxification response induced by HU treatment.

INTRODUCTION

Hydroxyurea (HU) is widely used as a reagent to promote replication fork stalling in a range of organisms (Krakoff et al., 1968; Young and Hodas, 1964). HU targets class I ribonucleotide reductase (RNR), the enzyme that catalyzes the formation of deoxyribonucleotides from ribonucleotides (Jordan and Reichard, 1998). Inhibition of RNR results in lowered cellular pools of deoxyribose nucleoside triphosphate (dNTPs) and thus leads to stalling of replication forks. Although all organisms possess RNR, the enzyme falls into distinct classes, classes I, II, and III. Class I RNRs further subdivide into classes Ia and Ib and are composed of a large R1 subunit and a small R2 subunit (Jordan and Reichard, 1998). A metal-containing reaction center in the R2 subunit generates a tyrosyl radical that is transferred to a cysteine in the R1 subunit, thereby generating a thiyl radical that, in turn, activates the substrate. HU acts to scavenge the tyrosyl radical, thereby inhibiting the reaction. The basis whereby inhibition of class I RNR leads to cell death has been established in *Escherichia coli* by work from Walker and colleagues (Davies et al., 2009). Depletion of dNTP pools leads to replication fork stalling. This triggers induction of the MazF and RelE toxins that in turn lead to improper translation of proteins and consequent membrane stress. Perturbation of terminal cytochrome oxidases leads to an increase in superoxide production. Upon superoxide conversion to hydrogen peroxide, the reaction of hydrogen peroxide with free ferrous iron leads to hydroxyl radical generation via the Fenton reaction. This effect is likely exacerbated by an influx of iron, triggered by a response to the requirement to synthesize increased levels of RNR (Davies et al., 2009). In addition to HU's action via the class I RNRs, it has been revealed that HU and its breakdown products can have a range of additional effects on cells. Kuong and Kuzminov (2009) have revealed that HU breaks down in aqueous solution to form nitrous oxide, cyanide, and peroxides, leading to the proposal that these compounds may contribute to the toxicity of HU.

In contrast to the class I RNRs, class II RNRs have a single subunit and generate their thiyl radical by cleavage of an adenosylcobalamin co-factor. Class II RNRs are not inhibited by HU. Class III enzymes are inhibited by oxygen and are restricted to obligate and facultative anaerobes (Jordan and Reichard, 1998). Interestingly, hyperthermophilic archaea of the genus *Sulfolobus* encode a class II RNR but also possess an open reading frame related to the NrdB R2 small subunit of a class I RNR (She et al., 2001). We were therefore intrigued to determine whether HU treatment of *Sulfolobus* had a physiological effect. How archaea deal with stalled replication forks is essentially unknown. Although the core archaeal DNA replication machinery is fundamentally related to that of eukaryotes, the majority of eukaryotic DNA repair checkpoint signaling and cell cycle regulators are not conserved between archaea and eukaryotes (Barry and Bell, 2006). Archaea possess orthologs of Rad51 (termed RadA in archaea), Rad50 and Mre11 and also the Hel308 helicase (Woodman and Bolt, 2009). Hel308 (also called Hjm) is conserved between archaea and metazoa but, curiously, is absent from yeast. It is a superfamily II helicase, and extensive biochemical and structural studies with mammalian and

This is an open access article under the CC BY license (http://creativecommons.org/licenses/by/4.0/).

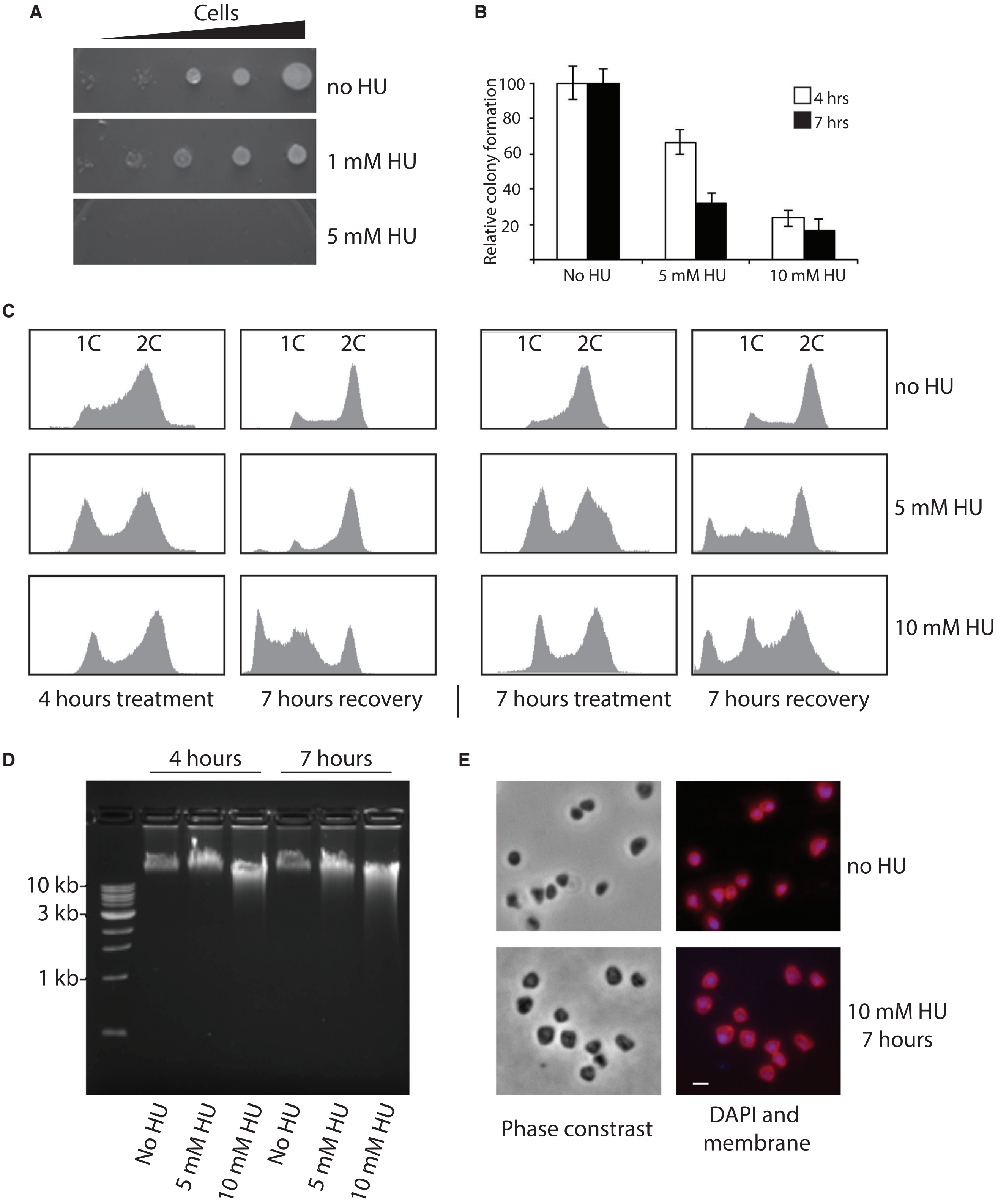

(legend on next page)

archaeal Hel308 orthologs have revealed it to be a potent helicase in vitro, adept at unwinding synthetic oligonucleotide replication fork substrates that contain a model nascent lagging strand. However, the precise range of activities observed seems to vary between different species and laboratories. Heterologous genetic assays have revealed that expression of archaeal Hel308 in an *E. coli dnaE486* strain resulted in synthetic lethality, essentially phenocopying the effect of expressing the *E. coli* fork regression helicase recQ in this background (Guy and Bolt, 2005). The *E. coli dnaE486* strain has a mutation in the α-subunit of DNA pol III that leads to elevated levels of stalled forks. These data therefore implicate Hel308 in interaction with stalled forks. However, Hel308 is essential for viability in archaea, and thus its physiological role in archaeal cells remains enigmatic (Woodman and Bolt, 2009; Zhang et al., 2013).

In the current work, we demonstrate that treatment of *Sulfolobus solfataricus* cells with HU leads to dose-dependent accumulation of DNA double-strand breaks and increases in early S-phase cell populations. Strikingly, we observe no robust decreases of dNTP pools. Both two-dimensional (2D) agarose gel electrophoresis and whole-genome marker frequency analyses reveal that replication initiation still occurs following low doses of HU treatment, but the rate of fork progression is impacted upon. Monitoring the levels of replication, chromatin, cell division and repair-associated proteins, and their transcripts reveals a subset of proteins to be selectively lost following HU treatment. In particular, we demonstrate that HU has a specific and direct effect on the DNA primase. We observe elevated levels of X-shaped DNA-junction-containing molecules, correlating with enhanced chromatin association of Hel308 and RadA following HU treatment. Finally, RNA-sequencing (RNA-seq) analyses reveal the induction of a set of genes suggestive of an anti-oxidant and detoxification response in *Sulfolobus*.

RESULTS

Sulfolobus Growth Is Inhibited by HU Treatment

Members of the hyperthermophilic archaeal genus *Sulfolobus* encode a gene, annotated as *nrdB*, encoding a protein homologous to the class I RNR small subunit (open reading frame SSO2498 in the *S. solfataricus* genome). However, the putative RNR small subunit gene shows a very restricted phyletic distribution within the archaea, being found in a subset of the *Sulfolobales* and some Halobacteria of the euryarchaea (Figure S1). In contrast, the sole *Sulfolobus* large-subunit RNR homolog (SSO0929) appears most closely related at the primary sequence level to class II and is conserved across the archaeal domain including many lineages that lack the R2-like proteins (Figure S1). It was therefore unclear whether *Sulfolobus* RNR would be sensitive to treatment of cells with HU.

First, we plated serial dilutions of *Sulfolobus solfataricus* P2 on gelrite plates containing increasing concentrations of HU. As seen in Figure 1A, chronic exposure to 5 mM HU completely inhibited growth. Next, we sought to determine the effect of acute exposure to HU on cell survival. We incubated exponentially growing *S. solfataricus* cells with 5 or 10 mM HU for 4 or 7 hr (Figure 1B). HU treatment was clearly toxic to cells with 66% cells remaining viable after 4 hr treatment with 5 mM HU, dropping to 24% after 7 hr exposure. 10 mM HU showed a stronger effect with 32% and 17% cells viable after 4 and 7 hr, respectively.

Next, we evaluated the effect of HU treatment on cell cycle phase distribution in the population (Figure 1C). A dose-dependent response could be observed with increasing one chromosome (1C) populations and elevated S-phase populations discernable by 5 mM HU; at 10 mM HU, we observed yet further elevation of the 1C population and the appearance of a population of cells with less than 1C genome content as adjudged by the shoulder appearing on the left slope of the 1C peak. Next, we tested the ability of *S. solfataricus* to recover from acute HU treatment. The cells treated with 0, 5, or 10 mM HU for 4 or 7 hr, were then pelleted, washed, resuspended in fresh medium, and grown for 7 hr, and cell cycle progression was monitored by flow cytometry (Figure 1C). Following recovery from a 4-hr treatment with 5 mM HU, the majority (>90%) of the population showed a normal cell cycle profile. However, treatment with 5 mM HU for 7 hr or 10 mM HU for either 4 or 7 hr resulted in appearance of enhanced signals of <1C content material, indicative of extensive cell death in the cultures. Significantly, DNA recovered from cells revealed a loss of integrity, with a smear of lower molecular weight material, indicative of accumulation of double-strand breaks, appearing following treatment with 10 or 5 mM HU for 7 hr (Figure 1D).

UV irradiation of *Sulfolobus* has also been shown to give rise to DNA damage; however, the cellular response to UV appears to be distinct from that to HU treatment (Götz et al., 2007). UV exposure leads to the gradual accumulation of cells with greater than two chromosome content and also induces a clumping response due to the induction of pili that appear to facilitate DNA transfer between cells (Ajon et al., 2011; Fröls et al., 2008). In contrast, we observe a HU dose-dependent decrease of DNA content and fail to detect any significant alterations in gross cell morphology or clumping following HU treatment (Figure 1E).

HU Treatment Leads to Alteration in Levels of a Subset of DNA Replication and Cell Division Proteins

Given the perturbation to the cell cycle profile of the population following HU treatment, we profiled relative levels of a number

Figure 1. Effects of Chronic and Acute Treatment of *Sulfolobus solfataricus* with Hydroxyurea

(A) Serial dilutions of *S. solfataricus* cells were plated on media containing the indicated concentrations of HU.

(B) Viable cell counts measured by plating efficiency following the indicated doses of HU; assays were performed in triplicates and the bars indicate the mean; error bars are SDs. Results are expressed relative to untreated cells (set at 100%).

(C) Flow cytometry profiles of cells following the indicated treatment and recovery times.

(D) Agarose gel analysis of the integrity of genomic DNA isolated from cells following exposure to 5 or 10 mM HU for 4 or 7 hr.

(E) Representative micrograph showing phase contrast and fluorescence imaging of treated or untreated cells. DNA was stained with DAPI and membranes with FM-464.

of DNA replication, DNA repair, and cell division-associated proteins following HU treatment. Initially, we tested the effect of 7-hr exposure to 10 mM HU (Figures 2A and 2B). Many of the proteins that we tested showed no significant changes in level following HU treatment (Figure 2A). These included replication fork-associated proteins such as the single-strand DNA binding protein (SSB); the replicative helicase MCM; the MCM-interacting factor Gins23; sliding clamp subunits PCNA1, 2, and 3; DNA ligase; replicative DNA polymerase polB1; and the clamp loader RFC. In addition, levels of WhiP, a homolog of the eukaryal pre-replicative complex protein Cdt1 that acts as the initiator protein that governs *oriC3* (Robinson and Bell, 2007; Samson et al., 2013); the chromatin protein Alba (Bell et al., 2002); the candidate fork regression helicase Hel308; and the recombinase RadA (the RAD51/RecA ortholog) were unaltered in their levels following treatment with HU.

However, a number of proteins were less abundant after treatment (Figure 2A); these included the replication initiators, Orc1-1 and Orc1-3, and the cell division protein Vps4 (Samson et al., 2008). The antisera that we have raised against the DNA primase recognized both catalytic subunit, PriS, and the PriL accessory subunit (Lao-Sirieix and Bell, 2004). Although levels of the catalytic subunit were unaltered, the regulatory subunit was highly reduced following treatment. This initial screen was performed with treatment by 10 mM HU for 7 hr, which results in significant cell death in the culture. We therefore tested the effect of 7-hr exposure of varying concentrations of HU on the levels of primase, Orc1-1 and Orc1-3 (Figure 2C). Selective depletion of Orc1-1, Orc1-3, and the regulatory subunit of DNA primase can be seen at 5 mM HU. Notably, the effect on Orc1-3 was more severe than that on Orc1-1 with minimal levels reached at 5 mM HU for Orc1-3. We also tested the effect of varying the time of exposure to 10 mM HU (Figure 2D). We could observe depletion of these proteins by 5 hr, preceding the accumulation of cell debris.

Next, we tested the effect of 4-hr treatment with 5 mM HU on the levels of transcripts for Orc1-1, Orc1-2, and Orc1-3, the regulatory subunit of primase, WhiP, RadA, Vps4, and Mre11 (Table S1). The transcript levels for WhiP, the protein levels of which remain unchanged, are similarly unaltered. For all the other genes, transcript levels mirror protein levels with the important exception of RadA, which, although unchanged at the protein level, shows 2.4-fold elevation of mRNA levels.

HU Alters the Replication Profile of *Sulfolobus*

Given the roles of the Orc1 proteins in defining replication origins and mediating replication initiation, and primase's role in synthesizing the primer for DNA synthesis, we were interested to determine the impact of HU treatment on the replication profile of cells. First, we profiled the global status of replication using marker frequency analysis (MFA). In this technique, DNA is quantified across the chromosome by measuring sequence tag abundance in 8-kbp windows by next-generation sequencing. The ratio of tag abundance from DNA purified from asynchronous replicating cells is then normalized to that of a non-replicating stationary phase culture. As can be seen in Figure 3A, replication origins are represented as peaks and termination zones as troughs in the resultant plots. Overlaying the plots for control cells and cells treated with 5 or 10 mM HU reveals that the amplitude of the profiles seen with 5 mM HU is higher than in control cells, indicating that a greater proportion of the cell cycle is spent in S-phase. The relative height of the *oriC2* peak is reduced compared to those for *oriC1* and *oriC3* (compare with the control panel). Treatment with 10 mM HU resulted in the peak corresponding to WhiP-dependent *oriC3* having the highest amplitude; in addition, the slopes of the peaks appear less uniform when compared to either control or 5 mM HU-treated samples, suggesting a less uniform rate of progression of replication forks within the cell population.

We next performed 2D neutral-neutral agarose gel analyses to assess firing at the origins (Figure 3B). Replication initiation arcs can be seen at *oriC1*, *oriC2*, and *oriC3* in control cells (0 mM HU). We can still detect these initiation structures at *oriC1* and *oriC3* and, extremely faintly, at *oriC2* in cells that have been treated with 5 mM HU for 4 hr. However, cells exposed to 10 mM HU for 4 hr no longer possess detectable bubble arcs. Instead, we now detect abundant species corresponding to X-shaped molecules at all three origins. To test whether these species were specific to origins, we performed 2D gels to examine replication structures at two further origin-distal loci (Figure 3C). At both of these regions, we also observe X-shaped structures following treatment with 10 mM HU.

HU Treatment Stimulates Hel308 and RadA Association with DNA

Next, we employed chromatin immunoprecipitation (ChIP) to test whether the residual Orc1 initiator proteins remain associated with origins following HU treatment. However, as can be seen in Figures 4A–4C, neither Orc1-1 nor Orc1-3 binds detectably to the origins following HU treatment. In contrast, WhiP, the levels of which are not affected by HU treatment, remains associated with *oriC3* (Figure 4C). Next, we performed ChIP analysis of the replicative helicase, MCM (Figure 4E). We observe up to 25-fold enrichment of MCM at both origin proximal and origin distal loci following HU treatment. This is not simply due to improved cross-linking or DNA recovery following HU treatment as ChIP testing the distribution of the chromatin protein Alba shows at most a 2.5-fold variation between treated and non-treated samples (Figure 4F). We propose therefore that the accumulation of MCM corresponds to elevated levels of stalled replication forks following HU treatment (Figure 3B). As discussed in the Introduction, biochemical studies have suggested that the essential Hel308 helicase may be involved in processing of stalled replication forks (Woodman and Bolt, 2009). We therefore performed ChIP analyses with antisera generated against this protein and observed an enrichment of up to 22-fold at origins of replication (Figure 4G). Interestingly, this protein was only modestly enriched (4.6-fold) at the non-origin locus where we observed 25-fold enrichment of MCM. Finally, we performed ChIP with the RecA/Rad51-like recombinase, RadA, and observed up to 7-fold enhancement of this protein at origins and distal loci upon HU treatment (Figure 4H).

HU Treatment Does Not Lead to Depletion of dNTP Pools

Thus, we observe HU-mediated toxicity and DNA damage as manifested by altered replication rates, increases in X-shaped

OPEN ACCESS
CellPress

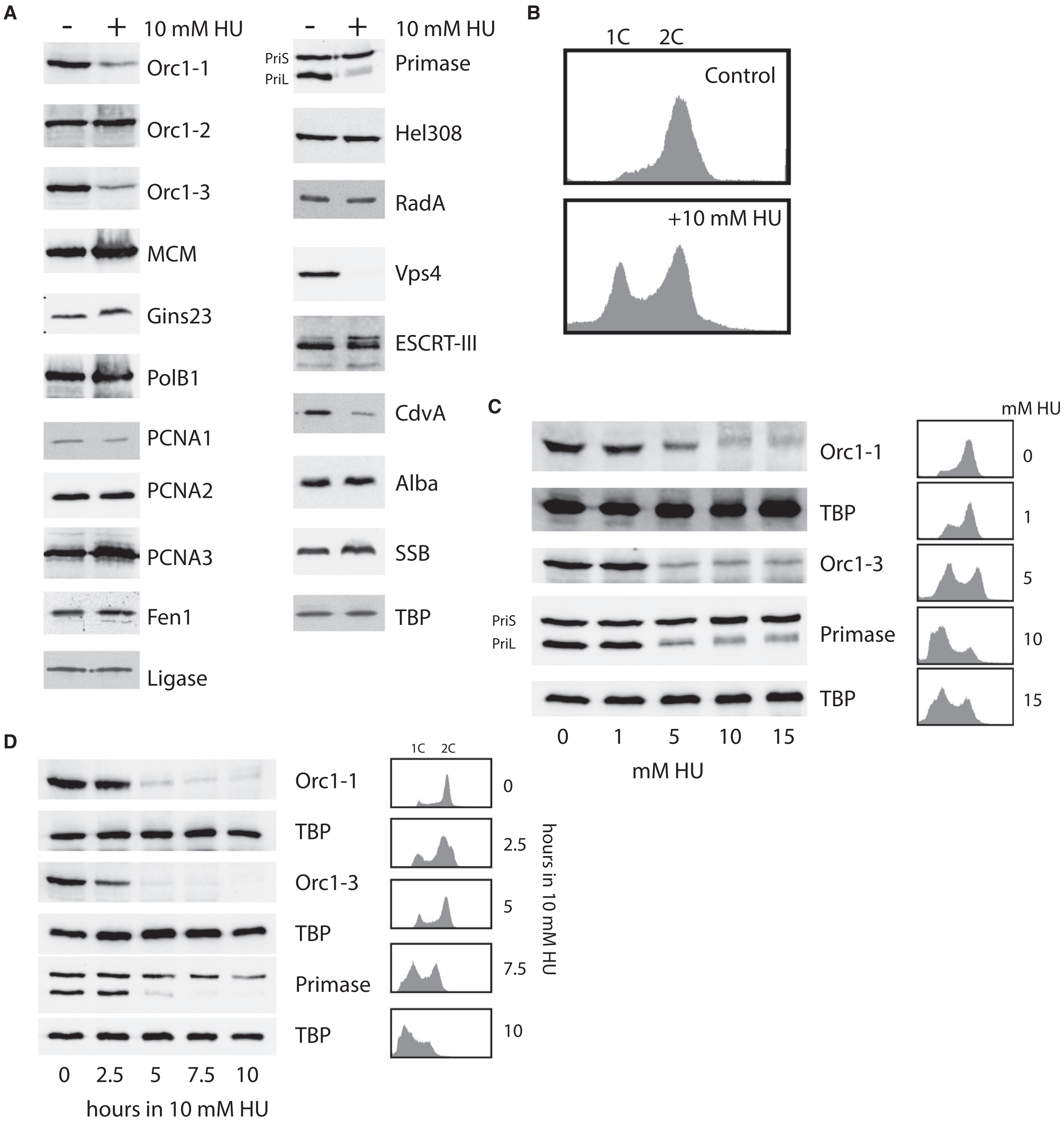

Figure 2. Molecular Consequences of Treatment of *S. solfataricus* with HU

(A) Western blot analysis of the levels of a variety of replication and cell division-associated proteins in *Sulfolobus* following treatment with 10 mM HU for 7 hr. The anti-WhiP antisera detects full-length protein and an additional truncated form. Loading for all panels was confirmed by western blotting for the general transcription factor, TBP. A single representative TBP panel is shown.

(B) Flow cytometry profile confirming the cell cycle arrest upon HU treatment for the cells used in (A).

(C) Western blot analyses of the effect of 7 hr exposure to the indicated concentrations of HU on the levels of Orc1-1, Orc1-3, and primase subunits. Flow cytometry profiles of the cells following treatment are shown on the right. TBP serves as a loading control in this and the subsequent panel.

(D) Effect of varying the time of exposure to 10 mM HU on the levels of Orc1-1, Orc1-3, and primase subunits. Flow cytometry profiles of the treated cells are shown to the right of the western blot images.

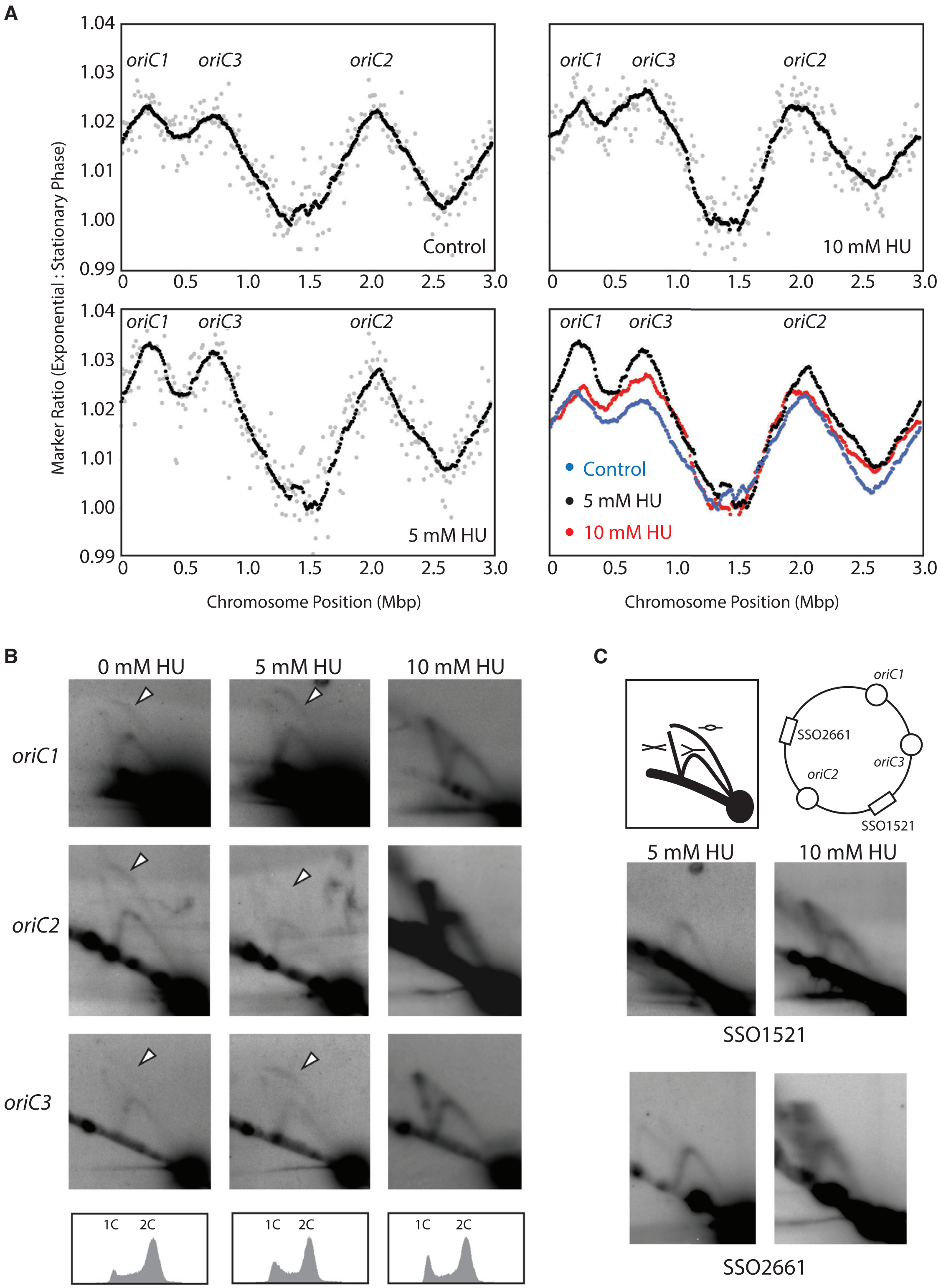

Figure 3. Perturbation of DNA Replication upon Treatment with HU

(A) Marker frequency analyses of the replication profile of cells treated with 0, 5, or 10 mM HU for 7 hr. DNA was recovered from the indicated cultures and subjected to next-generation sequencing on an Illumina platform. Read counts were binned into 8-kb windows and normalized to DNA isolated from G_2 (stationary phase) cells. Raw values for the individual counts are shown in gray, and a smoothed, moving 25-point average is shown in black. The lower right-hand panel shows an overlay of the three smoothed signals.

(legend continued on next page)

recombination structures, loss of DNA integrity, elevated chromatin association of the RadA recombinase and putative fork regression helicase, Hel308, and perturbation to the cell cycle profile with an increase in G_1/early S-phase cells. These phenotypes are reminiscent of the consequences of HU-mediated inhibition of RNR in eukaryotic cells with consequent depletion of dNTP pools leading to replication fork arrest. We therefore quantified dNTP levels in cells before and after treatment with 5 or 10 mM HU for 4 and 7 hr (Figure 5). Absolute levels of dNTPs and NTPs were measured. Levels of both dNTPs and NTPs drop with HU concentration and treatment time, likely in line with the loss of cell viability we observe following HU treatment. NTP levels serve as a normalization control for intact cells in the population. When the ratios of dNTP:NTPs were calculated, a modest (2- to 3-fold) increase in relative levels of all dNTPs apart from dCTP is observed. As treatment of *Sulfolobus* with HU results in cell cycle arrest in G_1/early S-phase, we propose that the increase of dATP, dGTP, and dTTP reflects the reduced rate of dNTP incorporation into DNA, thereby elevating pools of the precursors. In light of the lack of depletion of the other deoxyribonucleoside triphosphates, the drop in dCTP levels was surprising. Although this is a modest effect, at most 2-fold when normalized to NTP levels, we do not exclude the possibility that it may have an impact on the replication rate. The basis of the dCTP reduction is unresolved at this time, although we speculate that may be due to the action of dCTP deaminase in *Sulfolobus*.

Based on the presence of a vitamin B12-dependent, HU-insensitive, class II RNR in archaea, the substitution of the crucial tyrosine targeted by HU in the NrdB-like protein by phenylalanine, the grouping of the NrdB-like SSO2498 with the non-RNR-associated R2lox family of ligand-dependent oxidases, and the increase in relative levels of dATP, dGTP, and dTTP in HU-treated cells, we conclude that the primary mode of action of HU in *Sulfolobus* is not through inhibition of RNR.

Walker and colleagues (Davies et al., 2009) have revealed that HU induces hydroxyl-radical-mediated cell death in bacteria. This is thought to be effected in part by increased iron uptake leading to enhanced OH• formation via the Fenton reaction. Importantly, addition of the OH• scavenger thiourea to the medium prevented HU-mediated cell death of *E. coli* cultures. In contrast, however, thiourea had no impact on the cytotoxic effect of HU on *S. solfataricus* (Figure 5D). Additionally, we tested for induction of oxidative stress in *Sulfolobus* by monitoring the levels of *Ss*Dps (Maaty et al., 2009; Wiedenheft et al., 2005). The *Ss*Dps protein forms a cage-like structure with a di-iron-binding motif and facilitates the oxidation of Fe(II) to Fe(III). Importantly, although treatment of *S. solfataricus* cultures with 30 μM hydrogen peroxide results in the previously observed elevation of Dps levels (Figure 5E and see below), treatment with HU has no detectable impact on the levels of the *Ss*Dps protein.

Specificity of Primase Regulatory Subunit Depletion by HU

The data above indicate that initiation of DNA replication at all three origins is impacted by HU treatment, even though levels and chromatin association of WhiP, the initiator for *oriC3*, are unaffected by administration of HU. Our observation that the regulatory subunit of primase, PriL, is selectively lost upon HU treatment could account for the loss of origin firing and cessation of fork progression that we observe. However, it is formally possible that the effects we see on PriL levels could be a consequence of cell death, rather than a direct effect of HU treatment. To address this concern, we first subjected cells to two other genotoxic insults, UV treatment and treatment with hydrogen peroxide. We exposed cells to 200 J/m^2 UV, and then grew them for 4 or 7 hr before plating serial dilutions to test for viability (Figure 6A). To prevent photo-reactivation, the cells were grown in the dark prior to plating (Götz et al., 2007; Fröls et al., 2007). Viability was essentially unaffected by 4-hr growth in the dark after UV irradiation; however, growth for 7 hr prior to plating resulted in survival dropping by two orders of magnitude. Despite the differences in viability, western blotting revealed no significant changes in the absolute or relative levels of primase catalytic and regulatory subunits (Figure 6A). Similarly, treatment of cells with 30 or 100 μM hydrogen peroxide for 4 or 7 hr impacted on viability (Figures 6B and 6C), with severity of impact on cell viability scaling with concentration and exposure time. This ranged from an approximately 10-fold reduction in the number of viable cells with a 4-hr treatment with 30 μM hydrogen peroxide to complete loss of viability with administration of 100 μM hydrogen peroxide. Regardless of the impact on viability, relative levels of primase subunits were unaltered by H_2O_2 (Figures 6B and 6C). Thus, the effects of HU on levels of PriL appear to be specific to treatment with this agent, not simply a consequence of cell death. To determine whether HU could directly impact primase stability, we tested the effect of hydroxyurea on the purified primase complex in vitro. As can be seen in Figure 6D, top panel, incubation of the heterotrimeric PriSLX complex with 5 or 10 mM HU for 4 hr leads to enhanced precipitation of the complex. Compared to the water-treated control, we observe a 16- or 20-fold enrichment of primase in the insoluble material for 5 and 10 mM HU, respectively. Incubation of recombinant MCM helicase or DNA polymerase PolB1 with HU had no discernable impact on the solubility of these proteins (Figure 6D, lower panels).

The Genome-wide Transcriptional Response to HU Treatment

We observe HU-mediated toxicity and DNA damage as manifested by altered replication rates, increases in recombination structures, elevated chromatin association of the RadA recombinase and putative fork regression helicase, Hel308, yet dNTPs are not reduced compared to untreated controls. We propose

(B) 2D neutral-neutral agarose gel analyses of the *oriC1-3* following treatment with 0, 5, or 10 mM HU for 7 hr (flow cytometry profiles shown at bottom). White triangles point to the "bubble" arc indicative of replication initiation.

(C) A cartoon of the migration of the indicated bubble-containing, Y-shaped and X-shaped species is shown in the upper left panel, and the upper right panel reveals the relative positions of the loci probed. The lower four panels show the impact of HU treatment on the DNA structures detectable at the non-origin SSO1521 and SSO2661 loci.

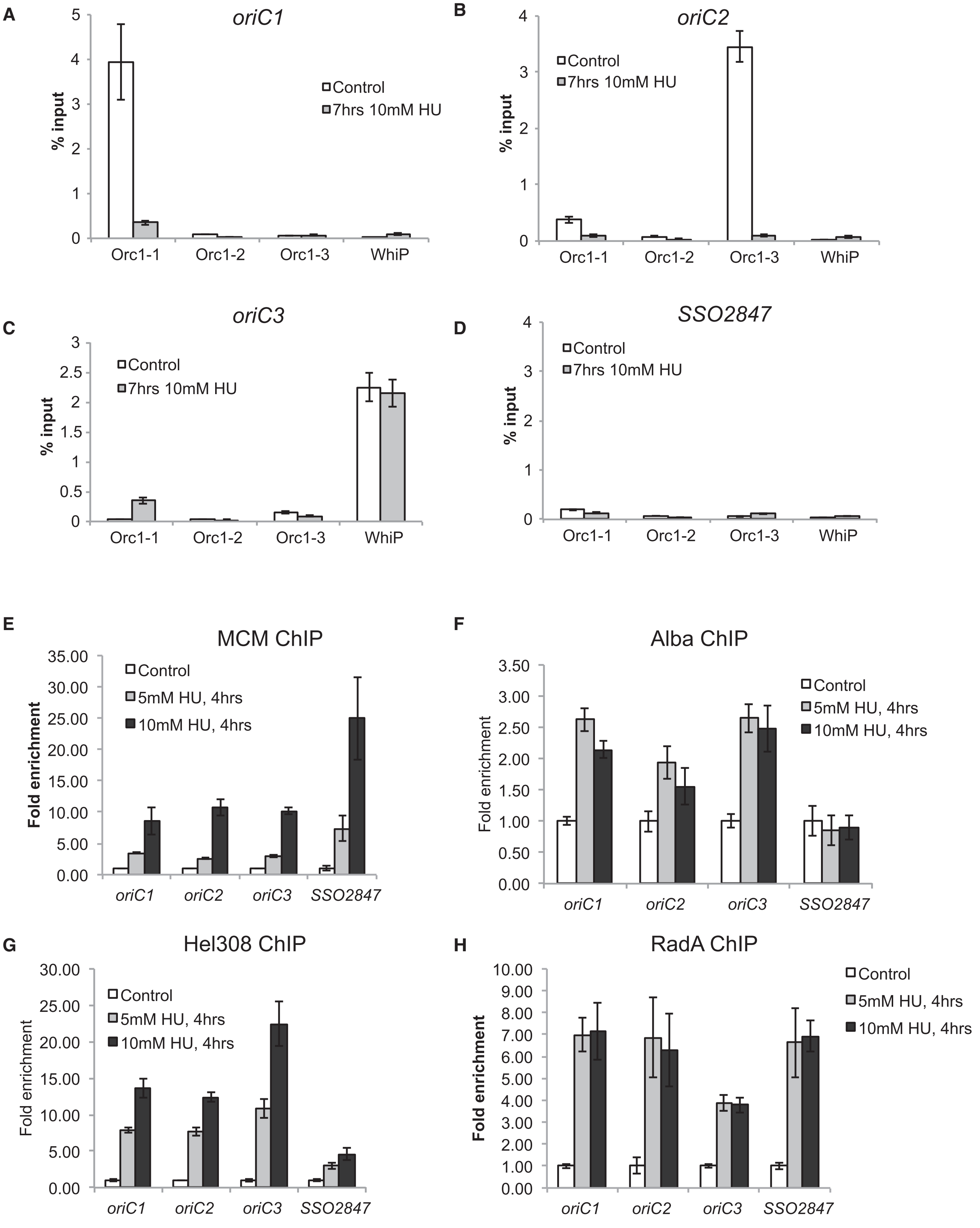

(legend on next page)

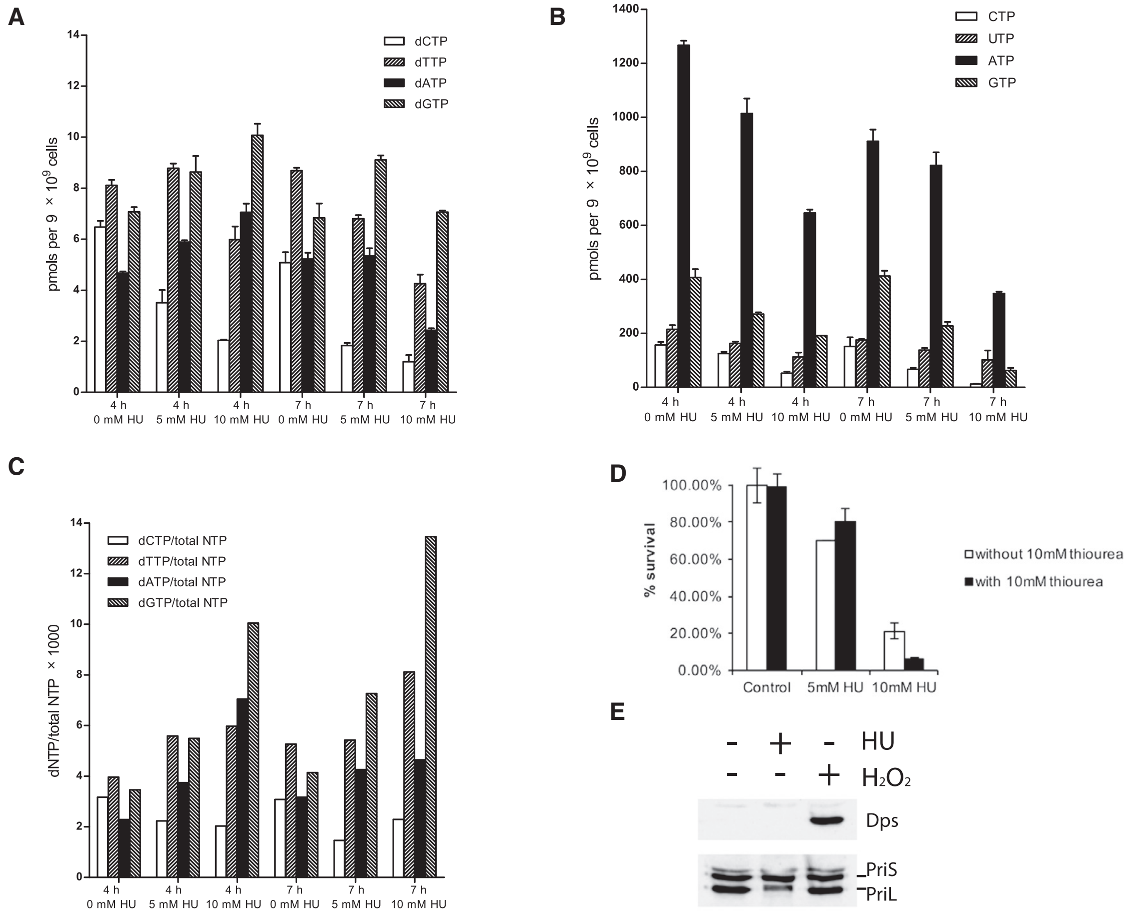

Figure 5. HU Treatment Does Not Decrease dNTP Pools

(A and B) dNTP (A) and NTP (B) levels were quantified following the treatment of cells with 0, 5, or 10 mM HU for 4 or 7 hr; measurements were performed in duplicate, and the bars indicate the mean; error bars are SDs.

(C) Mean dNTP pools normalized by the sum of all NTPs in each sample to account for cell death.

(D) HU toxicity is not ameliorated by addition of thiourea. Cells were grown for 4 hr with 0, 5, or 10 mM HU with (black) or without (white) 10 mM thiourea. Viable cell counts measured by plating efficiency following the indicated treatment; assays were performed in triplicate, and the bars indicate the mean; error bars are SDs. Results are expressed relative to untreated cells (set at 100%).

(E) Western blot analyses to test the consequences of hydrogen peroxide and hydroxyurea treatment on the levels of primase subunits and the Dps protein.

that loss of primase activity will contribute to the toxicity of HU but do not exclude the possibility that HU will have additional effects. To address the basis of the HU toxicity, we performed RNA-seq analyses on untreated cells and cells following exposure to 5 mM HU for 4 hr. There are no large-scale global changes in the overall transcript profile (Figure S4). Indeed, we only observe significant ($p < 0.05$) changes in the levels of 25 transcripts (Table 1). We observe a repression of genes involved in de novo purine biosynthesis. Conversely, thiamine synthesis genes, a number of genes encoding iron-binding proteins, antioxidant components, and a sulfur metabolism pathway are induced. The implications of these observations are discussed below.

DISCUSSION

In the current work, we have provided a description of the responses of a member of the archaeal domain of life to treatment with HU. The best-characterized role for HU is the inhibition of class I ribonucleotide reductase, and we were initially prompted

Figure 4. ChIP Analyses of Chromosome Occupancy by Replication Initiation and DNA Repair Factors Modulated by HU Treatment

(A–D) ChIP analyses of binding of the Orc1-3 and WhiP proteins to the three replication origins (A–C) and distal control locus SSO2847 (D) in cells treated with 0 or 10 mM HU for 7 hr. ChIP reactions were performed in triplicate, and data are expressed as fractional recovery of the total input material.

(E–H) Occupancy of the indicated genomic loci by MCM (E), Alba (F), Hel308 (G), and RadA (H) as adjudged by ChIP from untreated cells or cells treated with 5 or 10 mM HU for 4 hr.

Mean values of the triplicate repeats are shown, and error bars indicate the SD of the data.

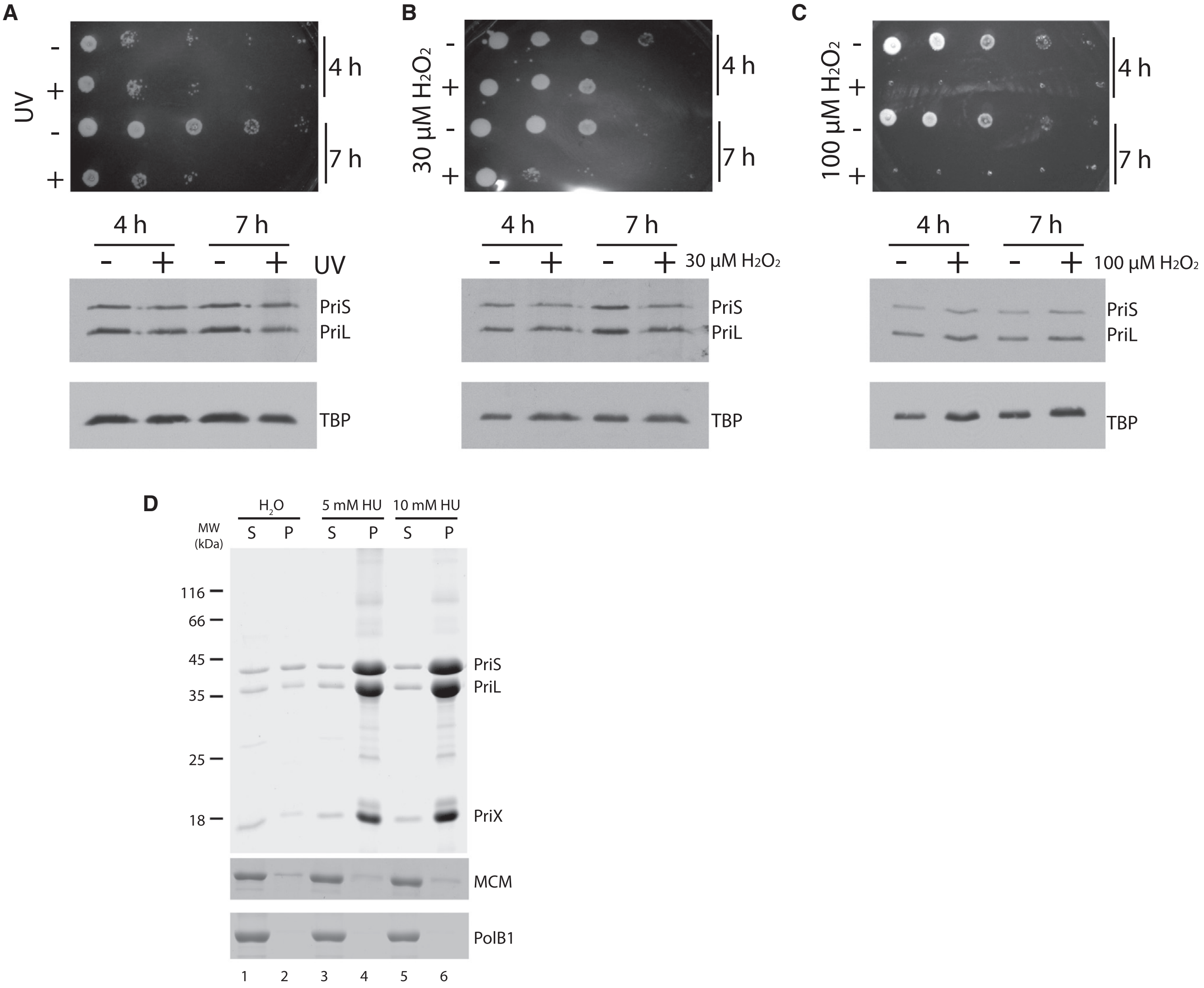

Figure 6. The Depletion of the Regulatory Subunit of Primase Is Specific to HU Treatment

(A–C) The upper panel shows plating of 10-fold serial dilutions either mock-treated (–) or treated (+) with (A) 200 J/m^2 of UV light (254 nm) or (B) 30 μM or (C) 100 μM hydrogen peroxide for the indicated times. The lower panels contain western blots to determine levels of primase and TBP proteins.

(D) Treatment of 13.5 μM recombinant primase (upper panel), MCM (middle panel), or PolB1 (bottom panel) with the indicated concentrations of HU for 4 hr at 78°C leads to selective precipitation of primase. Following treatment, samples were centrifuged to separate soluble (s) and precipitated (p) material and corresponding fractions analyzed by SDS-PAGE and stained with Coomassie brilliant blue.

to test the effect of HU on *Sulfolobus* because of the documented presence of a gene encoding a homolog of the class I RNR small subunit, NrdB. Furthermore, a number of recent studies have employed HU as a DNA-damaging agent in *Sulfolobus*, based on the untested assumption that it induces fork stalling, as in eukaryotes (Huang et al., 2015; Liang et al., 2013; van Wolferen et al., 2015). However, the RNR large subunit is clearly of the class II, vitamin B12-dependent, and HU-insensitive family. Regarding the NrdB homolog, recent work has described the existence of a series of NrdB-related proteins, termed R2lox (Andersson and Högbom, 2009; Högbom, 2011). This family was first identified in *Mycobacterium*, and although they have no demonstrated function, they are thought to play roles as ligand-dependent oxidases. One of the hallmarks of the R2lox family is the presence of a phenylalanine in place of the pivotal tyrosine residue in true RNR small-subunit proteins. Importantly, the *Sulfolobus* NrdB-like protein possesses a phenylalanine at this position. Furthermore, phylogenetic analyses clearly place it in the R2lox clade (Figures S2 and S3). Thus, it appears that this archaeal protein is not in fact a true RNR subunit. We also note that we have failed to detect any physical interaction between the *Sulfolobus* NrdB and NrdJ proteins. In agreement with the proposal that the NrdB-like protein is not a true RNR component, we demonstrate that there is no reduction of cellular dATP, dTTP, or dGTP pools following HU treatment. Intriguingly, we do observe a reduction of up to

Table 1. Transcripts Showing Significant ($p < 0.05$) Alterations in Level following Treatment with 5 mM Hydroxyurea for 4 hr

Genes	Description	Fold Change (log_2)	p Value
SSO2292*	Amino acid transport	−4.5	0.006
SSO6264*	Purine biosynthesis operon	−4.2	0.024
SSO0626*	5-Aminoimidazole-4-(*N*-succinylcarboxamide) ribonucleotide (SAICAR) synthase—and linked genes	−4	0.024
SSO3189*	Amino acid transporter	−3.9	0.019
SSO2017+8	Enoyl-CoA hydratase, 1,2-phenylacetyl-CoA epoxidase—detoxification—has di-iron center SSO2018 may have role in benzoate catabolism	3.3	0.049
SSO1817	Thiosulfate sulfur transferase	3.4	0.047
SSO2908:2909: 2911	Uroporphyrin-III C-methyltransferase (sirohemesynthase) (cysG): sulfite reductase hemoprotein beta component(cysl): 3′-phosphoadenosine 5′-phosphosulfatesulfotransferase (PAPS reductase) (cysH)—4Fe•4S cluster-containing	3.4	0.046
SSO3059	Maltose transporter, permease	3.4	0.046
SSO3058	Maltose transporter, permease	3.6	0.036
SSO2881	FeS-containing radical SAM enzyme	3.6	0.036
SSO2858 and 2860	Selenium binding protein (possible role in redox modulation)	3.7	0.036
SSO2912: 2913:2914	Sulfate adenylyltransferase (sat): hypothetical: TauE (sulfite or taurine exporter)	3.6	0.032
SSO1591	Symporter	3.6	0.036
SSO2019–2022	Benzoate catabolism	3.8	0.047
SSO2966	Hypothetical	3.8	0.046
SSO1369	Thiamine pyrophosphate-dependent dehydrogenase	3.9	0.036
SSO3045–3048	Oligopeptide transport	4	0.048
SSO2671	Oligopeptide ABC transporter	4	0.032
SSO2976	Hypothetical	4.2	0.024
SSO1593	Major facilitator superfamily transporter for benzoate	4.4	0.006
SSO1102	Hypothetical transcriptional regulator—possible metal binder	4.5	0.014
SSO1816	FeS-containing protein—ferredoxin-like	4.5	0.014
SSO3055	Maltose transporter, ABC	4.5	0.0061
SSO2089	Ten-A/THI-4—adjacent to protease, so more like TenA?	4.5	0.024
SSO1741+2	DoxAD thiosulfate:quinone oxidoreductase	4.7	0.0075
SSO1324	Thiamine biosynthesis—4Fe•4S-containing	4.7	0.0047

Genes showing reduced expression after HU treatment are highlighted with an asterisk (*).

2-fold in the levels of dCTP relative to total NTP levels. Although it is possible that this reduction in dCTP could impinge on replication rate, we note that similar reductions of dTTP in budding yeast have no impact on S-phase progression (Kohalmi et al., 1991; Liskay et al., 2007).

Although the classical target for HU is absent from archaea, HU treatment is clearly highly toxic to *Sulfolobus* cells, eliciting a graded series of responses depending on the concentration and timing of HU treatment. At 5 mM HU, we see ongoing replication initiation at *oriC1* and *oriC3* and reduced levels of initiation at *oriC2*, in the context of an overall slowing of S-phase, as detected both by flow cytometry and marker frequency analyses. We have previously shown that *oriC2* has a broader temporal window of firing within the cell population (Duggin et al., 2008). Our data could therefore indicate that impaired fork progression from *oriC1* and *oriC3* following HU treatment is sensed and results in a negative feedback to *oriC2*. This hypothesis is supported by the increased occupancy of genomic loci by MCM and a clear stimulation of the presence of the putative fork regression helicase Hel308 and the recombinase RadA on DNA, correlating with our observations of the accumulation of X-shaped DNA molecules at both origin-containing and non-origin loci. We note that Hel308 and RadA are essential for viability in *Sulfolobus*, preventing us from testing whether deletion of these genes sensitizes the organism to HU treatment (Zhang et al., 2013). Concomitant with these effects, we observe a reduction in the transcript levels for the Orc1-1 and Orc1-3 initiator proteins, and the regulatory subunit of primase. Conversely, the transcripts for RadA and Mre11 are elevated. Interestingly, although the operon encoding the genes for Rad50 and Mre11 has been previously shown to be upregulated by UV-induced DNA damage, the *radA* gene remained constitutively expressed after UV treatment (Fröls et al., 2007; Götz et al., 2007).

At 10 mM HU doses, we observe cessation of replication initiation at all three origins. For *oriC1* and *oriC2*, this can be explained by the marked reduction of the cognate Orc1-1 and

Orc1-3 initiator proteins (Robinson et al., 2004; Samson et al., 2013), and the consequent inability to detect Orc1-1 and Orc1-3 at origins by ChIP. However, levels of the WhIP protein, the initiator required for *oriC3*, are not altered, and ChIP reveals that this protein remains associated with *oriC3* even after treatment with 10 mM HU. Thus, at this origin, replication is being inhibited downstream of origin specification. We note that the inability to mediate replication initiation at all three origins correlates with the maximal loss of the regulatory subunit of primase, and we propose that this may be a principal cause of the replication arrest.

The selective loss of PriL, the regulatory subunit of DNA primase, is intriguing. We were concerned that the effects on primase could be indirect, particularly because we observe cell death in the culture upon HU treatment. However, lethal doses of either hydrogen peroxide or UV light do not result in any detectable loss of PriL, suggesting that either HU itself or a peculiarity of the death caused by HU impacts specifically on primase. In support of the proposal that the effect of HU is direct, we observe that primase is destabilized by treatment with HU in vitro. PriL contains a labile 4Fe•4S cluster in its C-terminal domain and thus could be highly sensitive to the redox status of cells (Klinge et al., 2007). We note that 5 of the 21 transcripts induced upon 5 mM HU treatment encode proteins with candidate iron-binding modules. We cannot, however, detect any evidence for induction of the oxidative stress response protein *Ss*Dps, in contrast to the effect observed upon treatment of cells with high (30 μM) concentrations of hydrogen peroxide. Furthermore, oxidative stress induction by H_2O_2 treatment does not lead to any discernable loss of the regulatory subunit of primase. Additionally, we do not observe any significant alteration in levels of transcripts for the genes encoding components of the SUF pathway that performs iron-sulfur cluster biosynthesis (Iwasaki, 2010). We note that hydroxyurea has been demonstrated to chelate iron ions in weakly acidic conditions (Konstantinou et al., 2011). This suggests a potential mechanism for the destabilization of the labile FeS cluster in PriL (Klinge et al., 2007). We speculate that destabilization of the cluster leads to localized unfolding of the PriL protein and thus accounts for the precipitation of the primase complex that we observe in vitro and targeted degradation of the destabilized PriL in vivo.

Although loss of the primase could account for the replication arrest we observe, it is likely that HU will have additional toxic effects in the cell. Kuzminov and colleague (Kuong and Kuzminov, 2009) revealed that "aged" HU solutions contain cyanide, nitrous oxide, and a number of peroxides. Notably, our RNA-seq data reveal the induction of a transporter for a free radical scavenger, benzoate, as well as a cluster of genes involved in benzoate catabolism. We additionally observe induction of biosynthetic enzymes for thiamine, an antioxidant, as well as elevated transcription of a sulfate utilization operon that encodes enzymes in a pathway that proceeds via adenosine 5′-phosphosulfate (APS) and sulfite to generate hydrogen sulfide. Notably, the gene for the sulfite reductase is upregulated, as is that for Uroporphyrin-III C-methyltransferase, responsible for biosynthesis of the siroheme co-factor necessary for sulfite reductase activity. Furthermore, we observe the induction of SSO1817, a thiosulfate sulfur-transferase and homolog of Rhodanese, an enzyme that de-toxifies cyanide utilizing thiosulfate to generate thiocyanate and sulfite (Cipollone et al., 2007). Elevated sulfite, itself a toxic metabolite, can be dealt with in three ways, via the action of sulfite reductase to generate hydrogen sulfide; via APS reductase and sulfate adenylate transferase; and finally by export from the cell via the sulfite exporter. Genes for proteins in all three of these pathways are upregulated in our RNA-seq dataset (Table 1; Figure S6).

We speculate therefore that, although we prepared fresh HU solutions prior to experiments, in accordance with the Arrhenius equation the high temperatures required to grow *Sulfolobus* (75–80°C) may have accelerated the generation of these toxic breakdown products, resulting in induction of the protective responses that we document in the RNA-seq analyses. Thus, we conclude that the extensive DNA damage we observe may be a consequence of direct action of these by-products rather than via depletion of dNTP pools and consequent replication fork stalling. In addition, destabilization of the iron-sulfur cluster in the regulatory subunit of the primase, leading to degradation of this subunit, will lead to cessation of DNA synthesis.

In addition to the reduction in levels of the subset of "early" replication factors, we observe reduced levels of the cell division protein CdvA and almost complete loss of the ATPase Vps4 (Samson et al., 2008, 2011). Notably, like Orc1-1 and Orc1-3, levels of Vps4 protein do not vary in a normal cell cycle, suggesting the existence of a targeting system that directs these proteins for degradation upon cellular stress (Duggin et al., 2008; Samson et al., 2008). However, unlike previous work where we have impaired cell division by overexpressing a *trans*-dominant-negative allele of Vps4, in the present situation we do not observe the generation of cells with a greater than 2C content (Samson et al., 2013). Thus, the combination of impairing replication initiation with targeting the cell division machinery appears to prevent overreplication events. This behavior could be viewed as a checkpoint response, allowing the cell time to repair damage. Given that *Sulfolobus* species lack the sophisticated kinase cascades that govern eukaryotic cell cycle progression, it will be of considerable interest to determine the nature and molecular basis of the coordinate regulation of eukaryotic-like core replication and cell division machineries.

EXPERIMENTAL PROCEDURES

Cell Growth and Drugs Treatment

S. solfataricus P2 cells were grown at 78°C in Brock's medium, pH 3.2, containing 0.1% (w/v) tryptone. Cells to be treated with HU (Sigma), thiourea (Sigma), or H_2O_2 (Sigma) were grown to A_{600} = 0.3 before an indicated amount of the drug was added. All drug treatments were done at 78°C. For HU block and release, a 20-mL HU-treated culture was washed three times with 2 mL of pre-warmed water before growth was resumed in fresh Brock's medium. For hydrogen peroxide and UV treatment, *S. solfataricus* P2 was grown in Brock's medium to an OD_{600} of 0.1–0.2 and then split into two cultures, and one was exposed to 200 J/m^2 UV using a SpectroLinker XL-1000 UV Crosslinker (Spectronics Corporation). For hydrogen peroxide treatment at 30 and 100 μM, hydrogen peroxide was added to one culture at 100 or 30 μM, whereas the other had water added in equal volume as a control. The liquid cultures were grown shaking at 78°C for 4 and 7 hr. At both time points, OD_{600} readings and cell extract samples for western blots were taken, and each culture was spotted onto plates as previously described.

Flow Cytometry

100 μL of culture was mixed with 700 μL of ice-cold ethanol and stored at 4°C. Cells were centrifuged for 5 min at 13,000 rpm and resuspended in 1 mL of 10 mM Tris-Cl (pH 7.4), 10 mM $MgCl_2$. Cells were centrifuged and then resuspended in the same buffer containing 10 μM Sytox Green (Invitrogen) and 100 μg/ml RNase A. Samples were analyzed on a Dako CyAn ADP flow cytometer with a 488-nm laser excitation, operated by Summit software.

Cell Viability Tests

Cell culture was diluted to A_{600} = 0.1 (equivalent to 1 × 10^8 cells/ml) with prewarmed water and was then 10-fold serially diluted to A_{600} = 1 × 10^{-4} (equivalent to 1 × 10^5 cells/ml). 50 μL of the diluted cells were plated onto Brock's plates in triplicate. Plates were incubated at 78°C until colonies appeared. The number of viable cells per ml was calculated by multiplying the average number of colonies formed on a plate by the dilution factor and dividing it by the volume of cells plated. For spotting assays, the cell culture was diluted to A_{600} = 0.1 (equivalent to 1 × 10^8 cells/ml) and then serial diluted in 10 folds concentration steps. 5 μL of each dilution were spotted onto gelrite plates made with varying concentrations of HU.

Whole-Cell Lysate Preparation and Western Blotting

20 mL of cell culture were harvested and resuspended in 20 μL of water per μg of pellet. SDS-PAGE loading buffer was added to 1×, and the sample was boiled for 5 min. Ten to 15 μL of whole-cell extract was separated by SDS-PAGE, and standard western blotting was then performed. Hel308 antibodies were raised in rabbits injected with purified recombinant *Sulfolobus* Hel308 protein (a kind gift from Malcolm White, St. Andrews, UK).

dNTP and NTP Pools Quantitation

A total of 2.7 × 10^{10} cells per measurement was harvested by filtration through 0.45 μm nitrocellulose filters (Millipore), resuspended in 0.7 mL of ice-cold 10% TCA and 15 mM $MgCl_2$, and processed as described (Jia et al., 2015).

Genomic DNA Preparation and Analysis

10 mL cell culture were harvested and resuspended in 300 μL of lysis buffer (20 mM Tris-HCl [pH 8], 5 mM EDTA, 1% SDS). The lysate was extracted at least three times with phenol:chloroform, 1:1, until the aqueous layer was clear. DNA was then ethanol precipitated and RNase A (1 mg/ml) digested. 5 μg DNA were analyzed by 1% agarose gel electrophoresis.

qRT-PCR and RNA-Seq

Total RNA purification, reverse transcription, and qPCR were carried out as described (Samson et al., 2008). Primers used for qPCR are listed in Table S2. RNA-seq was performed on RNA pooled from triplicate cultures of treated and untreated cells. Library preparation and sequencing were performed by the Wellcome Trust Centre for Human Genetics (Oxford, UK). Data were analyzed using the SeqMonk package (http://www.bioinformatics.babraham.ac.uk/projects/seqmonk/); significant changes in transcript levels were identified using the Intensity Difference method.

ChIP, qPCR, MFA, and 2D Agarose Gel Electrophoresis

MFA was performed as described; 2D gel electrophoresis and Southern transfer were carried out as detailed previously (Robinson et al., 2004). Probes for detecting Sso2661 and Sso1521 regions were amplified by PCR from *S. solfataricus* P2 genomic DNA using the primers listed in Table S3. ChIP and qPCR using the primers listed in Table S3 were carried out as described (Duggin et al., 2008). Primers used for qPCR are listed in Table S3.

Statistical Analysis

ChIP and qRT-PCR experiments were performed in triplicate, and SDs were calculated. RNA-seq was performed on pooled triplicate biological samples. Statistical analysis of the data was performed using the intensity difference test (http://www.bioinformatics.babraham.ac.uk/training/Advanced%20SeqMonk.pdf).

Cloning and Purification of PriSLX

PriX (Liu et al., 2015) was PCR amplified from *S. solfataricus* genomic DNA using two primers: PriX5′ (5′-GATCCCATATGAGTCAAGAGAAAAAAGCCAAAAAAATT) and PriX3′ (5′-GTCCTACCTCGAGTTAGCTATTTTTTAATACTCTTATTATCTCATTG), which contain the underlined restriction enzyme sites for NdeI and XhoI, respectively. The PriX gene was then inserted into the RSFDuet-1 vector (Novagen) to create the expression vector PriX-pRSF. PriS and PriL were previously cloned and expressed in our laboratory (Lao-Sirieix and Bell, 2004; expression vector-pETpri). *E. coli* Rosetta (BL21) cells were co-transformed with pETpri and PriX-pRSF and grown in LB medium supplemented with chloramphenicol, ampicillin, and kanamycin. When A_{600} reached 0.4, 1 mM isopropyl-β-D-thiogalactopyranoside was added to induce expression of PriS, PriL, and PriX. Additionally, the media was supplemented with 0.5 mM L-cysteine and 0.5 mM ammonium iron(II) sulfate hexahydrate to promote iron-sulfur cluster production in PriL. After 4 hr, cells were harvested by centrifugation. In order to limit iron sulfur cluster oxidation, the entire purification was carried out in a single day. Cells were lysed in buffer A (10 mM Tris [pH 8], 150 mM NaCl, 14 mM 2-mercaptoethanol) and a cocktail of protease inhibitors (Roche; Complete) by French press and pelleted. The supernatant was heat treated at 70°C for 20 min before a final centrifugation step. The remaining soluble supernatant was loaded onto a 5-mL HiTrap Heparin column (GE Healthcare) and eluted using a linear gradient from buffer A to buffer B (10 mM Tris [pH 8], 1 M NaCl, 14 mM 2-mercaptoethanol). Peak fractions eluted around 60% buffer B and were pooled and loaded onto a Superdex 26/600 75 (GE Healthcare) equilibrated with buffer A. The resulting peak fractions were pooled and flash frozen.

Precipitation Assay

100 μL reactions containing the indicated proteins at 13.5 μM were incubated with either water, 5 mM HU, or 10 mM HU. Reactions were incubated at 78°C (the growth temperature of *Sulfolobus*) for 4 hr. 60 μL of the whole reaction were pelleted, and 30 μL of the soluble fraction were mixed with loading dye. The pellet was resuspended in 30 μL of water and mixed with loading dye. Soluble and insoluble fractions were separated on an SDS-PAGE gel and stained with Coomassie dye. Enrichment of primase in the insoluble fractions was compared relative to the water-treated insoluble fraction and determined using the ImageQuant software.

ACCESSION NUMBERS

The accession number for the RNA-seq analysis reported in this paper is GEO: GSE87612.

SUPPLEMENTAL INFORMATION

Supplemental Information includes five figures and four tables can be found with this article online at http://dx.doi.org/10.1016/j.celrep.2016.10.024.

AUTHOR CONTRIBUTIONS

L.P.L., S.D.B., and A.C. designed experiments and interpreted the results. L.P.L., Z.Y.L., M.C., Z.K., and L.M. performed experiments. S.D.B. wrote the paper with input from all authors.

ACKNOWLEDGMENTS

We thank Malcolm White (St. Andrews, Scotland) for anti-RadA and anti-Alba antisera and for his generous gift of recombinant Hel308 protein. We also thank Jiangyu Yan and Yuli Xu for PolB1 and MCM, respectively. Thanks also to Mark Young for the anti-Dps antisera. We are grateful to Arnulf Kletzin, Bettina Siebers, and David Giedroc for helpful discussions regarding sulfur metabolism and Britt-Marie Sjöberg regarding RNR classes. L.P.L. was funded by the BBSRC, and Z.K. has a postdoctoral stipend from the Kempe Foundation. Work in S.D.B.'s laboratory in Oxford was funded by the BBSRC and Wellcome Trust and in Indiana by the College of Arts and Sciences, Indiana University. M.C. was supported by the Graduate Training Program in Quantitative

Chemical Biology under award number T32 GM109825 and Indiana University. The content is solely the responsibility of the authors and does not necessarily represent the official views of the NIH. Work in A.C.'s laboratory was funded by the Swedish Research Council and the Swedish Cancer Society.

Received: May 13, 2016
Revised: July 29, 2016
Accepted: October 7, 2016
Published: November 1, 2016

REFERENCES

Ajon, M., Fröls, S., van Wolferen, M., Stoecker, K., Teichmann, D., Driessen, A.J., Grogan, D.W., Albers, S.V., and Schleper, C. (2011). UV-inducible DNA exchange in hyperthermophilic archaea mediated by type IV pili. Mol. Microbiol. *82*, 807–817.

Andersson, C.S., and Högbom, M. (2009). A *Mycobacterium tuberculosis* ligand-binding Mn/Fe protein reveals a new cofactor in a remodeled R2-protein scaffold. Proc. Natl. Acad. Sci. USA *106*, 5633–5638.

Barry, E.R., and Bell, S.D. (2006). DNA replication in the archaea. Microbiol. Mol. Biol. Rev. *70*, 876–887.

Bell, S.D., Botting, C.H., Wardleworth, B.N., Jackson, S.P., and White, M.F. (2002). The interaction of Alba, a conserved archaeal chromatin protein, with Sir2 and its regulation by acetylation. Science *296*, 148–151.

Cipollone, R., Ascenzi, P., and Visca, P. (2007). Common themes and variations in the rhodanese superfamily. IUBMB Life *59*, 51–59.

Davies, B.W., Kohanski, M.A., Simmons, L.A., Winkler, J.A., Collins, J.J., and Walker, G.C. (2009). Hydroxyurea induces hydroxyl radical-mediated cell death in *Escherichia coli*. Mol. Cell *36*, 845–860.

Duggin, I.G., McCallum, S.A., and Bell, S.D. (2008). Chromosome replication dynamics in the archaeon *Sulfolobus acidocaldarius*. Proc. Natl. Acad. Sci. USA *105*, 16737–16742.

Fröls, S., Gordon, P.M.K., Panlilio, M.A., Duggin, I.G., Bell, S.D., Sensen, C.W., and Schleper, C. (2007). Response of the hyperthermophilic archaeon *Sulfolobus solfataricus* to UV damage. J. Bacteriol. *189*, 8708–8718.

Fröls, S., Ajon, M., Wagner, M., Teichmann, D., Zolghadr, B., Folea, M., Boekema, E.J., Driessen, A.J., Schleper, C., and Albers, S.V. (2008). UV-inducible cellular aggregation of the hyperthermophilic archaeon *Sulfolobus solfataricus* is mediated by pili formation. Mol. Microbiol. *70*, 938–952.

Götz, D., Paytubi, S., Munro, S., Lundgren, M., Bernander, R., and White, M.F. (2007). Responses of hyperthermophilic crenarchaea to UV irradiation. Genome Biol. *8*, R220.

Guy, C.P., and Bolt, E.L. (2005). Archaeal Hel308 helicase targets replication forks in vivo and in vitro and unwinds lagging strands. Nucleic Acids Res. *33*, 3678–3690.

Högbom, M. (2011). Metal use in ribonucleotide reductase R2, di-iron, di-manganese and heterodinuclear—an intricate bioinorganic workaround to use different metals for the same reaction. Metallomics *3*, 110–120.

Huang, Q., Li, Y., Zeng, C., Song, T., Yan, Z., Ni, J., She, Q., and Shen, Y. (2015). Genetic analysis of the Holliday junction resolvases Hje and Hjc in *Sulfolobus islandicus*. Extremophiles *19*, 505–514.

Iwasaki, T. (2010). Iron-sulfur world in aerobic and hyperthermoacidophilic archaea *Sulfolobus*. Archaea *2010*, 842639.

Jia, S., Marjavaara, L., Buckland, R., Sharma, S., and Chabes, A. (2015). Determination of deoxyribonucleoside triphosphate concentrations in yeast cells by strong anion-exchange high-performance liquid chromatography coupled with ultraviolet detection. Methods Mol. Biol. *1300*, 113–121.

Jordan, A., and Reichard, P. (1998). Ribonucleotide reductases. Annu. Rev. Biochem. *67*, 71–98.

Klinge, S., Hirst, J., Maman, J.D., Krude, T., and Pellegrini, L. (2007). An iron-sulfur domain of the eukaryotic primase is essential for RNA primer synthesis. Nat. Struct. Mol. Biol. *14*, 875–877.

Kohalmi, S.E., Glattke, M., McIntosh, E.M., and Kunz, B.A. (1991). Mutational specificity of DNA precursor pool imbalances in yeast arising from deoxycytidylate deaminase deficiency or treatment with thymidylate. J. Mol. Biol. *220*, 933–946.

Konstantinou, E., Pashalidis, I., Kolnagou, A., and Kontoghiorghes, G.J. (2011). Interactions of hydroxycarbamide (hydroxyurea) with iron and copper: implications on toxicity and therapeutic strategies. Hemoglobin *35*, 237–246.

Krakoff, I.H., Brown, N.C., and Reichard, P. (1968). Inhibition of ribonucleoside diphosphate reductase by hydroxyurea. Cancer Res. *28*, 1559–1565.

Kuong, K.J., and Kuzminov, A. (2009). Cyanide, peroxide and nitric oxide formation in solutions of hydroxyurea causes cellular toxicity and may contribute to its therapeutic potency. J. Mol. Biol. *390*, 845–862.

Lao-Sirieix, S.H., and Bell, S.D. (2004). The heterodimeric primase of the hyperthermophilic archaeon *Sulfolobus solfataricus* possesses DNA and RNA primase, polymerase and 3′-terminal nucleotidyl transferase activities. J. Mol. Biol. *344*, 1251–1263.

Liang, P.J., Han, W.Y., Huang, Q.H., Li, Y.Z., Ni, J.F., She, Q.X., and Shen, Y.L. (2013). Knockouts of RecA-like proteins RadC1 and RadC2 have distinct responses to DNA damage agents in *Sulfolobus islandicus*. J. Genet. Genomics *40*, 533–542.

Liskay, R.M., Wheeler, L.J., Mathews, C.K., and Erdeniz, N. (2007). Involvement of deoxycytidylate deaminase in the response to S(n)1-type methylation DNA damage in budding yeast. Curr. Biol. *17*, R755–R757.

Liu, B., Ouyang, S., Makarova, K.S., Xia, Q., Zhu, Y., Li, Z., Guo, L., Koonin, E.V., Liu, Z.-J., and Huang, L. (2015). A primase subunit essential for efficient primer synthesis by an archaeal eukaryotic-type primase. Nat. Commun. *6*, 7300. http://dx.doi.org/10.1038/ncomms8300.

Maaty, W.S., Wiedenheft, B., Tarlykov, P., Schaff, N., Heinemann, J., Robison-Cox, J., Valenzuela, J., Dougherty, A., Blum, P., Lawrence, C.M., et al. (2009). Something old, something new, something borrowed; how the thermoacidophilic archaeon *Sulfolobus solfataricus* responds to oxidative stress. PLoS One *4*, e6964.

Robinson, N.P., and Bell, S.D. (2007). Extrachromosomal element capture and the evolution of multiple replication origins in archaeal chromosomes. Proc. Natl. Acad. Sci. USA *104*, 5806–5811.

Robinson, N.P., Dionne, I., Lundgren, M., Marsh, V.L., Bernander, R., and Bell, S.D. (2004). Identification of two origins of replication in the single chromosome of the Archaeon Sulfolobus solfataricus. Cell *116*, 25–38.

Samson, R.Y., Obita, T., Freund, S.M., Williams, R.L., and Bell, S.D. (2008). A role for the ESCRT system in cell division in archaea. Science *322*, 1710–1713.

Samson, R.Y., Obita, T., Hodgson, B., Shaw, M.K., Chong, P.L., Williams, R.L., and Bell, S.D. (2011). Molecular and structural basis of ESCRT-III recruitment to membranes during archaeal cell division. Mol. Cell *41*, 186–196.

Samson, R.Y., Xu, Y., Gadelha, C., Stone, T.A., Faqiri, J.N., Li, D., Qin, N., Pu, F., Liang, Y.X., She, Q., and Bell, S.D. (2013). Specificity and function of archaeal DNA replication initiator proteins. Cell Rep. *3*, 485–496.

She, Q., Singh, R.K., Confalonieri, F., Zivanovic, Y., Allard, G., Awayez, M.J., Chan-Weiher, C.C.Y., Clausen, I.G., Curtis, B.A., De Moors, A., et al. (2001). The complete genome of the crenarchaeon *Sulfolobus solfataricus* P2. Proc. Natl. Acad. Sci. USA *98*, 7835–7840.

van Wolferen, M., Ma, X., and Albers, S.V. (2015). DNA processing proteins involved in the UV-induced stress response of *Sulfolobales*. J. Bacteriol. *197*, 2941–2951.

Wiedenheft, B., Mosolf, J., Willits, D., Yeager, M., Dryden, K.A., Young, M., and Douglas, T. (2005). An archaeal antioxidant: characterization of a Dps-like protein from *Sulfolobus solfataricus*. Proc. Natl. Acad. Sci. USA *102*, 10551–10556.

Woodman, I.L., and Bolt, E.L. (2009). Molecular biology of Hel308 helicase in archaea. Biochem. Soc. Trans. *37*, 74–78.

Young, C.W., and Hodas, S. (1964). Hydroxyurea: inhibitory effect on DNA metabolism. Science *146*, 1172–1174.

Zhang, C., Tian, B., Li, S., Ao, X., Dalgaard, K., Gökce, S., Liang, Y., and She, Q. (2013). Genetic manipulation in *Sulfolobus islandicus* and functional analysis of DNA repair genes. Biochem. Soc. Trans. *41*, 405–410.

Cell Reports, Volume *17*

Supplemental Information

Hydroxyurea-Mediated Cytotoxicity

Without Inhibition of Ribonucleotide Reductase

Li Phing Liew, Zun Yi Lim, Matan Cohen, Ziqing Kong, Lisette Marjavaara, Andrei Chabes, and Stephen D. Bell

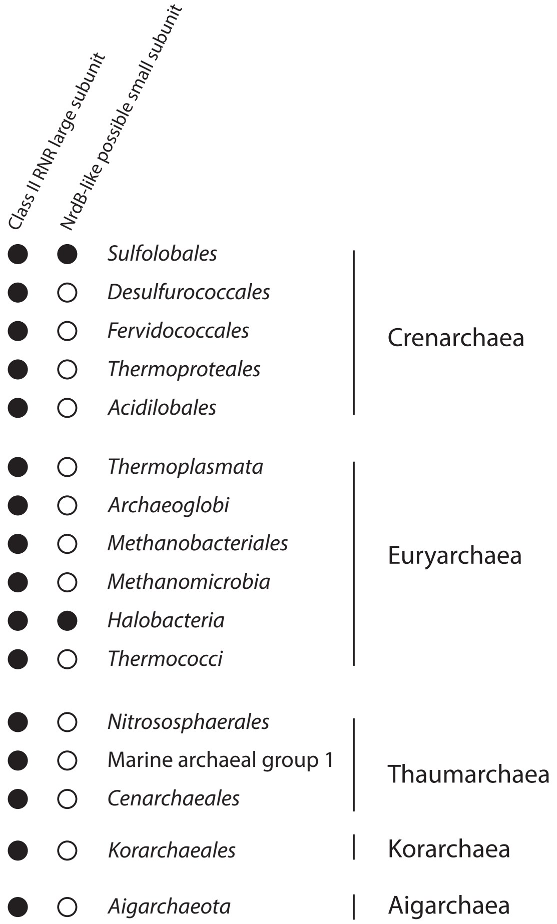

Figure S1. Distribution of Class II Ribonucleotide Reductase large subunits and homologs of the NrdB Class I small subunit in the indicated groups of archaea (related to Figure 1). Filled circles indicate presence of homologs, open circles indicate their absence.

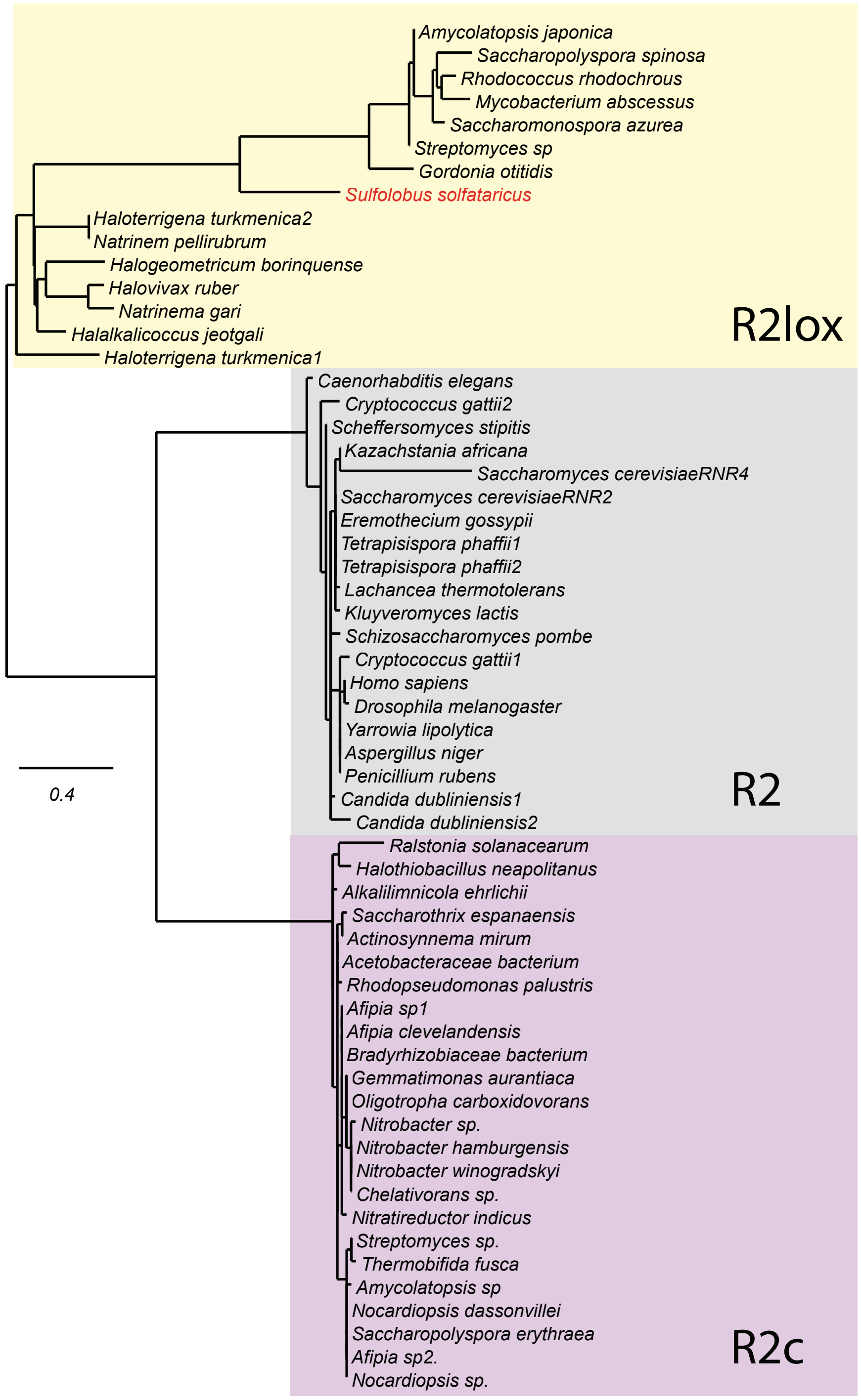

Figure S2. *Sulfolobus* "NrdB" is not a bona fide RNR-subunit but rather is a R2lox family member (related to Figure 1). Phylogenetic analyses of homologs of the NrdB-like protein reveal that the *Sulfolobus* SSO2498 gene product (red) is a member of the R2lox family of non-RNR-associated ligand-dependent oxidases (Andersson and Hogbom, 2009; Hogbom, 2011). The tree was generated using the server at www.phylogeny.fr (Dereeper et al., 2008). Accession numbers are listed in Table S4.

```
SSO2498        1 ------------------------------------------------------------
Ecoli          1 -MAYTTF-----------------------------------------------------
Pseudomonas    1 MLSWDEFDEEDTTTAAPATAAAQPASQTAVKLDNEAAGSVEEARAVAADDSDAIARAKRA

SSO2498        1 -----MGMSFE---------EYKHEYFKSIRS-----GGLNWSLFPMKLYQLGKKLFWDP
Ecoli          7 ------SQ--------TKNDQLKEPMFFGQPVNVARYDQQKY-DIFEKLIEKQLSFFWRP
Pseudomonas   61 LDELDIREGLNELEGEAARVRVDEKRMINARADLNQLVPFKY-DWAWQKYLDGCANHWMP

SSO2498       42 ANIDLSKDAEDWKKLNDLE----KMFIINVGSKFAAGEEAVALDLHPLIVTLVKEGRVEE
Ecoli         52 EEVDVSRDRIDY---QALPEHEKHIFISNLKYQ-TLLDSIQGRSP--NVALLPLISIPEL
Pseudomonas  120 QEVNMNADIALWKSKDGLSEDERRIVKRNLGFF-STADSLVANNL--VLATYRLITNPEC

                                   *
SSO2498       98 VMYLEQFVYEESKHVEAFRRFFDIVN-----------VMEDLSAYTKDLSPNYKKIFYEE
Ecoli        106 ETWVETWAFSETIHSRSYTHIIRNIVNDPSVVFDDIVTNEQIQKRAEGISS-----YYDE
Pseudomonas  177 RQYILRQAFEEAIHTHAYQYCIESLGMDEGEIFNMYHEIPSVAKK---------------

SSO2498      147 LP--KAMWNLSRDPS------------PE---NQVRAVVTYNLIVEGVAAEGGYNIFRQI
Ecoli        161 LIEMTSYWHLLGEGTHTVNGKTVTVSLRELKKKLY-LCLMSVNALEAIRFYVSFACSF-A
Pseudomonas  222 -----ASWGLKYT--RSISDPTFQTGTPETDKQFLKNLIAYYCVLEGIFFYCGFTQIL-S

SSO2498      190 TNTRKILPGLAKMVNLIATDESRHIAFGIYLITRLVKEYGEGV------------YKAAM
Ecoli        219 FAERELMEGNAKIIRLIARDEALHLTGTQHMLNLLRSGADDPEMAEIAEECKQECYDLFV
Pseudomonas  274 MGRRNKMTGTAEQFQYILRDESMHLNFGIDMINQIKIENPHLWDA----EMKDEATQMIL

SSO2498      238 DHINYLAPYAIGIFSEPTMPQVESLPFNLTNIELVDYAKKLLNTRIDAINRARRMKLEML
Ecoli        279 QAAQQEKDW-----ADYLFRDGSMI--GLNKDILCQYVEYITNIRMQAVGLDL------P
Pseudomonas  330 QGTQLEIEY-----ARDTMP-RGVL--GMNAAMMEDYLKFIANRRLTQIGLKE------E

SSO2498      298 LPKD---LDVIESW-------------------------------------
Ecoli        326 FQTRSNPIPWINTWLVSDNVQVAPQEVEVSSYLVGQIDSEVDTDDLSNFQL
Pseudomonas  376 YPGTTNPFPWMSEIMDLKKEKNF-FETRVIEYQTGGALS---WD-------
```

Figure S3 The *Sulfolobus* NrdB-related protein lacks the residue that forms the tyrosyl radical in true Class I RNR small subunits (related to Figure 1). Sequence comparison of *Pseudomonas aeruginosa* and *E. coli* NrdB with the *S. solfataricus* NrdB-like protein SSO2498. The essential tyrosine residue in the bacterial proteins is highlighted in red indicating its substitution by phenylalanine in the archaeon. Proteins sequences were aligned using ClustalOmega (Sievers et al., 2011) and shaded using the BoxShade Server (http://www.ch.embnet.org/software/BOX_form.html)

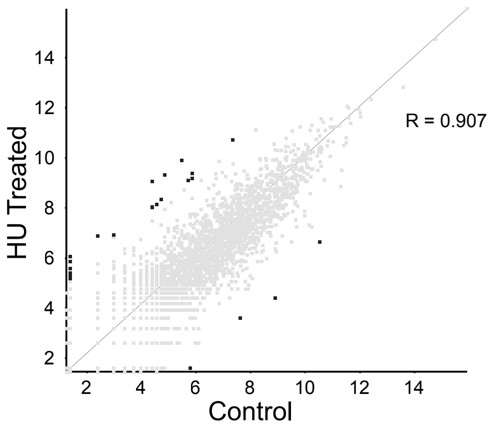

Figure S4. Comparison of RNA Seq data from control and treated cells (related to Table 1). Scatter plot comparing RNA-Seq results from HU-treated and untreated *S. solfataricus* cells. Statistically significant changes in level are indicated in black.

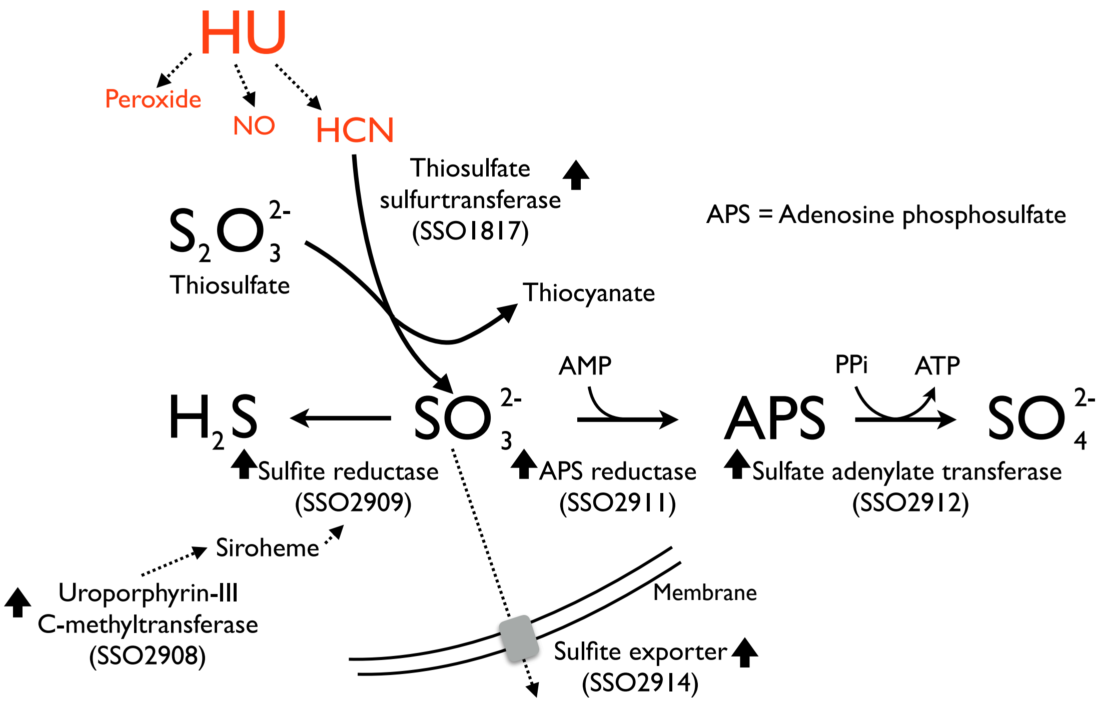

Figure S5. Potential detoxification pathway for HU breakdown products inferred from RNA Seq data (related to Table 1). RNA Seq data reveal elevated levels of transcripts for the enzymes indicated by the bold black arrows. We propose that cyanide generated by the thermal breakdown of HU is fed into this pathway by the action of thiosulfate sulfurtransferase generating thiocyanate and sulfite.

Gene	mRNA ratio (HU treated:untreated)
orc1-1	0.25
orc1-2	1.7
orc1-3	0.62
whiP	1.1
priL	0.64
radA	2.4
mre11	2.3

Table S1:Related to Figure 2: qRT-PCR quantitation of the indicated transcripts, comparing cells treated with 0 and 5 mM HU for 4 hours.

Oligonucleotide	Sequence (5' – 3')
Orc1-1fwd	GGGAAAGACAGCCGTAGTGA
Orc1-1rvs	AACCCGGTAAATGGAACCTT
Orc1-2fwd	GGATCGCCAGTGGTATTGTT
Orc1-2rvs	CCCATTGTGATCTCATCAGC
Orc1-3fwd	AGCACGGTGATGCAAGGAAAGCA
Orc1-3rvs	CACTAACGTGTCAACCTCGTCAAGA
WhiPfwd	CCCAGATCCAAATTGGTTGA
WhiPrvs	CGCTTAAGTGCCATCTTCCT
RadAfwd	TGGCCGTAAGGCAACAAAAGCT
RadArvs	ACCACCAACTGCCACAGTAGGGT
Mre11fwd	ACCACGACACTCCAAAGAGG
Mre11rvs	GCGGATAACGCGCTAACTAA
PriLfwd	TGAACCCGTACTCGTGTTTT
PriLrvs	TCTCCTCATTCTCGGTGTGA

Table S2, related to Figure 2: Primer pairs used for the qRT-PCR analyses.

Oligonucleotide	Sequence (5' – 3')
Sso1521fwd	GGCAAGTGGGTTGTTGAGAT
Sso1521rvs	TTACAGCGTCCCAAAAGTCC
Sso2661fwd	GGAGCCGCAGCAGTATTAAG
Sso2661rvs	ACGCATACCCTGAAAGATCG
OriC1fwd	TTTACCAGAGACCTACCCCATTGTTTC
OriC1rvs	GGGACCCATCTATTTCCTCTGG
OriC2fwd	TGTTTCAGATGAAATTAAGAGAGTGTCTC
OriC2rvs	CCCAGTTATGCAGTAAATTTCCTGTG
OriC3fwd	GTTATACTTCTATGGGCATATTGGGG
OriC3rvs	TGACATTAAATTCTCCCTCCTACTTGC
Sso2847fwd	GGGAAGCACTAAGCCAGATAATCC
Sso2847rvs	ACCTGAAATGCAGCTGGAGG

Table S3: Related to Figure 4. Primers used for amplifying the loci indicated in their names. The Sso1521 and Sso2661 yielded amplicons that were used for preparation of probes for 2D gel analyses. The remaining primers were used in qPCR quantitation of DNA recovered in ChIP experiments.

Accession Number	Species
AAK42633.1	*Sulfolobus solfataricus P2*
WP_008415238.1	*Halalkalicoccus jeotgali*
WP_006054776.1	*Halogeometricum borinquense*
WP_012945201.1	*Haloterrigena turkmenica ORF1*
WP_012945997.1	*Haloterrigena turkmenica ORF2*
WP_015298815.1	*Natrinema pellirubrum*
WP_008456675.1	*Natrinema gari*
AGB17208.1	*Halovivax ruber*
WP_052617888.1	*Mycobacterium abscessus*
WP_054370857.1	*Rhodococcus rhodochrous*
WP_005448065.1	*Saccharomonospora azurea*
WP_010308649.1	*Saccharopolyspora spinosa*
WP_007238417.1	*Gordonia otitidis*
WP_038507879.1	*Amycolatopsis japonica*
WP_009083906.1	*Streptomyces sp. AA4*
ABI57445.1	*Alkalilimnicola ehrlichii*
EHL99301.1	*Acetobacteraceae bacterium*
WP_012823881.1	*Halothiobacillus neapolitanus*
WP_011582020.1	*Chelativorans sp. BNC1*
EKS35359.1	*Afipia clevelandensis*
EGP10090.1	*Bradyrhizobiaceae bacterium SG-6C*
WP_009340611.1	*Afipia sp.1*
WP_009451442.1	*Nitratireductor indicus*
WP_013224744.1	*Afipia sp.2*
ABD89058.1	*Rhodopseudomonas palustris*
WP_009075962.1	*Streptomyces sp. AA4*
WP_016333204.1	*Amycolatopsis sp.*
ACI93866.1	*Oligotropha carboxidovorans*
WP_009947973.1	*Saccharopolyspora erythraea*
ABA06137.1	*Nitrobacter winogradskyi*
CCH32194.1	*Saccharothrix espanaensis*
BAH38790.1	*Gemmatimonas aurantiaca*
ABE64392.1	*Nitrobacter hamburgensis*
WP_014908832.1	*Nocardiopsis sp.*
WP_009796979.1	*Nitrobacter sp.*
ACU37069.1	*Actinosynnema mirum*
ADH69995.1	*Nocardiopsis dassonvillei*
WP_011293419.1	*Thermobifida fusca*
NP_982406.1	*Eremothecium gossypii*
XP_001401310.1	*Aspergillus niger*
NP_497821.1	*Caenorhabditis elegans*
XP_002418255.1	*Candida dubliniensis1*

XP_002418662.1	*Candida dubliniensis2*
XP_003196606.1	*Cryptococcus gattii1*
XP_003196693.1	*Cryptococcus gattii2*
XP_003959265.1	*Kazachstania africana*
XP_455757.1	*Kluyveromyces lactis*
XP_002551960.1	*Lachancea thermotolerans*
XP_002567793.1	*Penicillium rubens*
XP_001382334.2	*Scheffersomyces stipitis*
WP_003273165.1	*Ralstonia solanacearum*
NP_596546.1	*Schizosaccharomyces pombe*
XP_003685845.1	*Tetrapisispora phaffii1*
XP_003688271.1	*Tetrapisispora phaffii2*
XP_500466.1	*Yarrowia lipolytica*
AJR59499.1	*Saccharomyces cerevisiae RNR2*
NP_001025	*Homo sapiens*
NP_525111.1	*Drosophila melanogaster*
AJR77267.1	*Saccharomyces cerevisiae RNR4*

Table S4 Related to Figures 1 and S2: Accession numbers of proteins sequences used in the phylogenetic tree in Figure S2.

Additional References

Dereeper, A., Guignon, V., Blanc, G., Audic, S., Buffet, S., Chevenet, F., Dufayard, J.F., Guindon, S., Lefort, V., Lescot, M., Claverie, J.M. and Gascuel, O. (2008) Phylogeny.fr: robust phylogenetic analysis for the non-specialist. Nucleic Acids Res. *36*, W365-369

Sievers, F., Wilm, A., Dineen, D., Gibson, T.J., Karplus, K. ,Li, W., Lopez, R., McWilliam, H., Remmert, M., Söding, J., Thompson, J.D. and Higgins, D.G. (2011) Fast, scalable generation of high-quality protein multiple sequence alignments using Clustal Omega. Mol. Syst. Biol. *7,* 539
